# The Insulator Binding Protein CTCF Positions 20 Nucleosomes around Its Binding Sites across the Human Genome

**DOI:** 10.1371/journal.pgen.1000138

**Published:** 2008-07-25

**Authors:** Yutao Fu, Manisha Sinha, Craig L. Peterson, Zhiping Weng

**Affiliations:** 1 Bioinformatics Program, Boston University, Boston, Massachusetts, United States of America; 2 Interdisciplinary Graduate Program, University of Massachusetts Medical School, Worcester, Massachusetts, United States of America; 3 Program in Molecular Medicine, University of Massachusetts Medical School, Worcester, Massachusetts, United States of America; 4 Program in Bioinformatics and Integrative Biology, University of Massachusetts Medical School, Worcester, Massachusetts, United States of America; 5 Department of Biomedical Engineering, Boston University, Boston, Massachusetts, United States of America; Netherlands Cancer Institute, Netherlands

## Abstract

Chromatin structure plays an important role in modulating the accessibility of genomic DNA to regulatory proteins in eukaryotic cells. We performed an integrative analysis on dozens of recent datasets generated by deep-sequencing and high-density tiling arrays, and we discovered an array of well-positioned nucleosomes flanking sites occupied by the insulator binding protein CTCF across the human genome. These nucleosomes are highly enriched for the histone variant H2A.Z and 11 histone modifications. The distances between the center positions of the neighboring nucleosomes are largely invariant, and we estimate them to be 185 bp on average. Surprisingly, subsets of nucleosomes that are enriched in different histone modifications vary greatly in the lengths of DNA protected from micrococcal nuclease cleavage (106–164 bp). The nucleosomes enriched in those histone modifications previously implicated to be correlated with active transcription tend to contain less protected DNA, indicating that these modifications are correlated with greater DNA accessibility. Another striking result obtained from our analysis is that nucleosomes flanking CTCF sites are much better positioned than those downstream of transcription start sites, the only genomic feature previously known to position nucleosomes genome-wide. This nucleosome-positioning phenomenon is not observed for other transcriptional factors for which we had genome-wide binding data. We suggest that binding of CTCF provides an anchor point for positioning nucleosomes, and chromatin remodeling is an important component of CTCF function.

## Author Summary

The accessibility of genomic DNA to regulatory proteins and to the transcriptional machinery plays an important role in eukaryotic transcription regulation. Some regulatory proteins alter chromatin structures by evicting histones in selected loci. Nonetheless, no regulatory proteins have been reported to position nucleosomes genome-wide. The only genomic landmark that has been associated with well-positioned nucleosomes is the transcriptional start site (TSS)—several well-positioned nucleosomes are observed downstream of TSS genome-wide. Here we report that the CCCTC-binding factor (CTCF), a protein that binds insulator elements to prevent the spreading of heterochromatin and restricting transcriptional enhancers from activating unrelated promoters, possesses greater ability to position nucleosomes across the human genome than does the TSS. These well-positioned nucleosomes are highly enriched in a histone variant H2A.Z and 11 histone modifications. The nucleosomes enriched in the histone modifications previously implicated to correlate with active transcription tend to have less protected DNA against digestion by micrococcal nuclease, or greater DNA accessibility. This nucleosome-positioning ability is likely unique to CTCF, because it was not found in the other transcriptional factors we investigated. Thus we suggest that the binding of CTCF provides an anchor for positioning nucleosomes, and chromatin remodeling is an important aspect of CTCF function.

## Introduction

The positioning of nucleosomes along eukaryotic chromatin affects accessibility of the genomic DNA *in vivo*. Nucleosomes may bind to some genomic regions tightly and prevent transcription factors from approaching their sites. Alternatively, strategically positioned nucleosomes can promote long-range DNA bending and allow distal enhancers to interact with the transcriptional machinery [1]–[3]. Crystal structures show that each nucleosome contains 147 base-pairs (bp) of DNA tightly wrapped around an octamer of H2A, H2B, H3 and H4 histone proteins [4]. The linker DNA between two neighboring nucleosomes is ∼20 bp in *Saccharomyces cerevisiae* [5] and estimated to be 70 bp in higher eukaryotes [6]. Defined lysine and arginine residues in histone tails are often methylated and/or acetylated, which can recruit chromatin remodeling factors and regulate transcription. Histone variants prefer selected genomic regions, e.g. H2A.Z tends to flank nucleosome-free regions [7]–[10].

High resolution maps of nucleosome and H2A.Z have been generated for *S. cerevisiae* by subjecting chromatin to micrococcal nuclease (MNase) and detecting the undigested DNA with tiling arrays [11]–[13]. These studies revealed that RNA polymerase II promoters contain a nucleosome-free region of ∼200 bp upstream of the transcription start site (TSS), flanked by well-positioned nucleosomes on both sides. The same approach was used to map nucleosomes on a subset of human promoters [14]. Recently Zhao and colleagues generated a genome-wide nucleosome map using MNase digestion followed by deep sequencing (MNase-Seq) [15]. These studies confirmed the nucleosome-free region upstream of the TSS and several well-positioned nucleosomes downstream of the TSS in humans. In addition, the Zhao lab combined MNase digestion, chromatin immunoprecipitation, and deep sequencing to generate genome-wide maps of H2A.Z and 20 different types of histone methylation in humans [16].

Although the majority of occupied transcription factor binding sites are devoid of nucleosomes in yeast [11], little is known about how transcription factors and nucleosomes compete for genomic DNA in human cells. We integrated several genome-wide maps of transcription factor binding [16]–[19] and susceptibility of chromatin to DNase I [20] with the aforementioned nucleosome, H2A.Z, and histone modification maps [15],[16] to study this problem. We found that the insulator binding protein CTCF (CCCTC-binding factor) has an unusual ability to position multiple nucleosomes flanking its binding sites genome-wide.

CTCF has been extensively studied for its impact on imprinting and X-inactivation [21]. It binds to insulator elements to prevent the spread of heterochromatin and to restrict transcriptional enhancers from activating unintended promoters. In addition, it may function as a transcriptional repressor as well as an activator [22]–[24]. The DNA-binding domain of CTCF contains 11 zinc fingers. One study indicated that only 4 fingers are essential [25], while others showed that different combination of fingers are used to bind divergent sites [24],[26]. CTCF is thought to form special chromatin structures or mediate long-range chromosomal interactions in mammalian cells [24], [27]–[30]; however, the detailed mechanism remains unknown.

Our analysis led to several major findings: 1. CTCF binds in the center of a linker region, flanked by at least 20 well-positioned nucleosomes, symmetrically distributed around the CTCF binding side. We determined the extent that the TSS positions downstream nucleosomes with the same set of data, and were surprised to find that it is much less than that of CTCF. We also examined the genome-wide binding data of STAT1, NRSF, and p53, and found these factors to be incapable of positioning nucleosomes. 2. The nucleosomes flanking a CTCF site are highly enriched in H2A.Z and enriched in 11 histone modifications to various extents. 3. We determined that on average 150 bp of DNA in these nucleosomes is protected against MNase cleavage, and 35 bp of DNA is cleaved, although both quantities vary greatly among nucleosomes enriched in different histone variants or modifications. The two lengths for the same nucleosome are tightly anti-correlated, consistent with the nucleosome being well positioned. 4. The nucleosomes enriched in those histone modifications previously associated with active transcription tend to be less protected against MNase, suggesting greater DNA accessibility to the factors that regulate transcription. 5. CTCF protects roughly 60 bp of DNA and increases the linker between its two neighboring nucleosomes to 118 bp. 6. Sequence conservation was only observed for the CTCF binding site and not for the other positions in the surrounding 2 kb region, indicating that there is no evolutionary pressure on the genomic DNA sequence that positions the nucleosomes. Furthermore, a previously published algorithm predicts CTCF binding sites to be occupied by nucleosomes. Finally, we performed *in vitro* nucleosome mapping experiments on two insulator DNA fragments that each contains three CTCF sites. We found these CTCF sites to be located between MNase cleavage sites and hence are likely to be occluded by nucleosomes in the absence of CTCF. Thus we suggest that the binding of CTCF provides an anchor for positioning neighboring nucleosomes and this may be important for CTCF function.

## Results

### Aggregation of Mononucleosome Mapping and Histone Modification Data Reveal Well-Positioned Nucleosomes Flanking Occupied CTCF Sites

**Figure 1:**
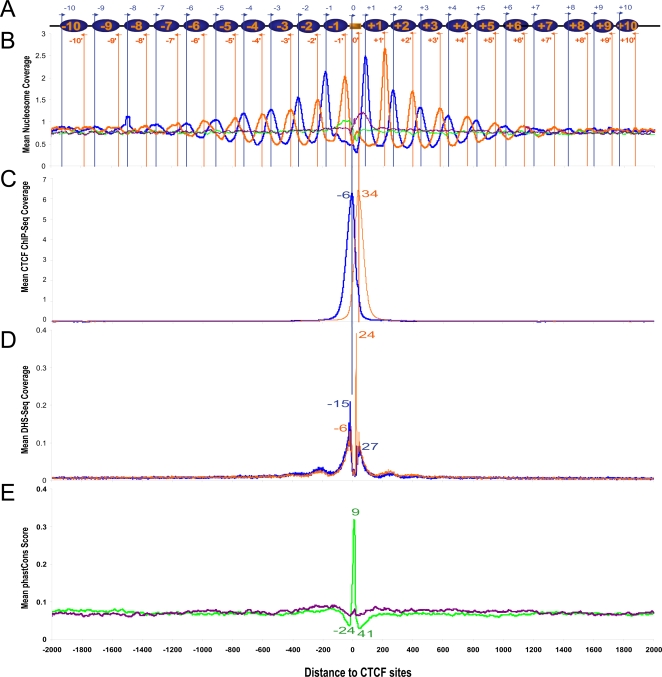
Aggregation of genomic signals around CTCF sites. The coordinate origin is set to the 5′-end position of the 20-bp-long CTCF site. Panel A shows the schematic arrangement of nucleosomes (blue ovals) around a CTCF binding site (orange rectangle). Blue arrows indicate sequence tags on the same strand as the CTCF site (plus strand), and orange arrows indicate opposite-strand (minus strand) tags. Panels B, C and D show the mean coverage of sequence tags for all mononucleosomes, CTCF ChIP-Seq and DNase-Seq around CTCF sites, respectively. The curves for *occupied* CTCF sites are colored blue for the plus strand tags and orange for minus strand. The green and purple curves in panel B represent plus strand and minus strand curves for *unoccupied* CTCF sites. Panel E shows the mean phastCons scores around occupied (green) and unoccupied (purple) CTCF sites. Note that the ChIP-Seq peaks are 12 bp inside the nucleosome boundaries as explained in Methods. The locations of the major peaks in all the panels are labeled.

We developed a method to perform aggregation analysis of genome-wide mapping data, called Genomic Signal Aggregator (GSA; see Methods and Figure S1). GSA computes distribution of hybridization score obtained in a tiling array experiment or coverage of sequence tags obtained in a deep sequencing experiment, plotted as a function of the distance to a set of anchors. We applied GSA to the deep sequencing data on mononucleosome mapping [15], with *occupied* CTCF binding sites (defined in Methods) as anchors. The average coverage of sequence tags for the DNA ends of mononucleosomes is shown in Figure 1B. We separately mapped the tags to the plus and minus strands (defined according to the strand of the anchor CTCF site), resulting in the blue and orange curves in Figure 1B (see Figure S2 for technical explanation on why there are two peaks per nucleosome). The CTCF site was observed in the center of a linker region, flanked on each side by up to 10 pairs of peaks with ∼185 bp intervals, indicating 20 well-positioned nucleosomes (Figure 1A). As a negative control, we aggregated the same data [15] but using *unoccupied* CTCF sites (defined in Methods) as anchors and produced two curves which peaked at +69 and −25 bp respectively, suggesting that unoccupied CTCF sites are often occupied by a nucleosome. Because the distance between these two peaks is smaller than the size of a nucleosome, we suspect that the nucleosome is positioned in slightly different positions across the CTCF sites.

In order to simulate the effect of sequencing depth, we generated two aggregation graphs with 20% and 5% of randomly sampled sequence reads from the original 154.6 M reads (Figure S3). Contrasting Figure S3 with Figure 1B indicates that greater sequencing depth leads to linearly taller aggregation graphs, such that the ratio of the sequence coverage around the occupied sites over the coverage around the unoccupied sites is largely independent of the sequencing depth. This ratio corresponds to how much more likely that a position, at a particular distance (<2 kb) away from an occupied CTCF site, is the end position of a mononucleosome, over the position anchored on an unoccupied CTCF site. In addition, greater sequencing depth leads to smoother graphs overall and in particular for the peaks that are far away from the occupied CTCF sites. As a result, more well-positioned nucleosomes are discernable with greater sequencing depth.

In Figure 1B, the peaks that are more distal from the anchoring CTCF site are broader and lower. To determine whether this indicates lower nucleosome occupancy in the distal regions, we integrated the sequence coverage over non-overlapping 185-bp intervals and computed the ratio between the resulting values for each interval anchored on occupied CTCF sites over the values for the same interval anchored on unoccupied CTCF sites. This resulted in a largely flat distribution with an average ratio of 1.07 (Figure S4), indicating that the nucleosome occupancy does not decrease appreciably over a 2 kb distance from an occupied CTCF site. Figure S4 further indicates that the nucleosome occupancy in the 4-kb region around occupied CTCF sites is 7% higher than that of the 4-kb region around unoccupied CTCF sites. The diminished and widened peaks distal from the CTCF anchors are likely due to the more distal nucleosomes being less well positioned across the cell population, and/or are positioned at more varying locations from the CTCF site among different CTCF-bound loci. The graphs for individual loci do not exhibit the diminishing behavior (not shown), suggesting that the distal nucleosomes are well-positioned across the cell population, but at more varying locations from the CTCF anchors than the proximal nucleosomes.

All of the experimental datasets were generated on the entire cell population, while CTCF could occupy its sites in a subpopulation of the cells. We generated aggregation graphs anchored on two subsets of occupied CTCF sites: sites that are 2–5 kb away from the nearest occupied sites and sites that are more than 500 kb away from the nearest occupied sites, hypothesizing that sites in the former set are more clustered and hence likely to be occupied in a greater portion of cells than sites in the latter set. Indeed a stronger signal was observed for the graphs anchored on the more clustered CTCF sites (Figure S5). Nonetheless, the difference between the two sets of graphs is small, suggesting that our findings are unlikely affected by the subpopulation issue.

**Figure 2:**
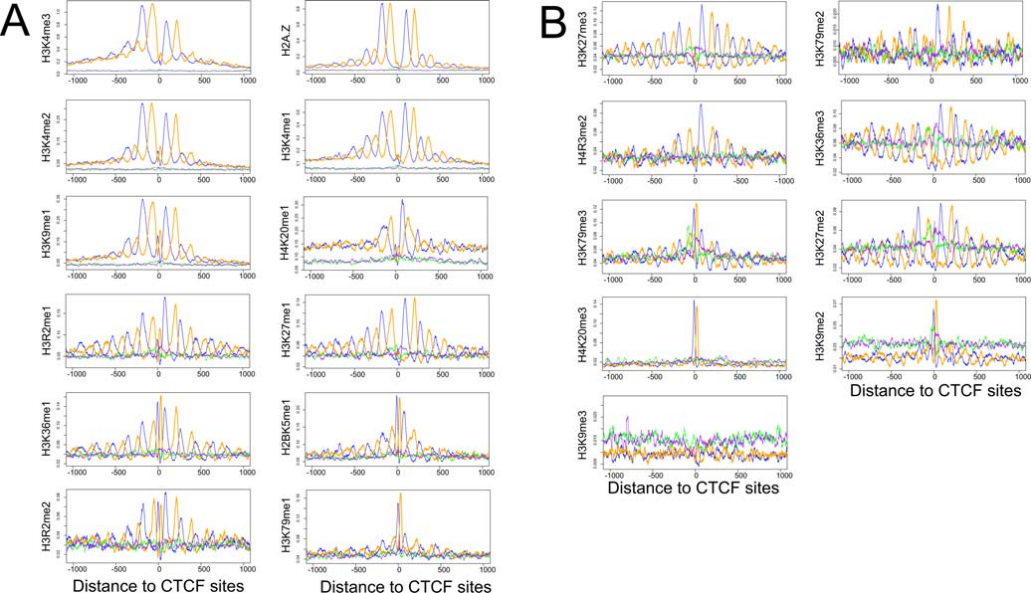
Aggregated ChIP-Seq tag coverage of 20 histone modifications and H2A.Z around CTCF binding sites. The blue and orange curves are for plus- and minus-strand tags around occupied CTCF sites, and green and purple curves are for plus- and minus-strand tags around unoccupied CTCF sites, respectively. A. Histone modifications enriched around occupied CTCF binding sites; B. Un-enriched histone modifications. Figures from top-left to bottom-right are sorted by descending level of enrichment over +/−2 kb.

We then applied GSA to all 20 ChIP-Seq datasets, each on mononucleosomes enriched in a type of histone modification [16]. With the exception of H3K9me3, the other 19 datasets show similarly dramatic oscillation (Figure 2). The H3K9me3 data may be of poor quality because it is not enriched around any of the anchor sets tested in this study (see the section after next for enrichment analysis). For 10 datasets (e.g., unmodified nucleosomes, or nucleosomes with modified H3K36, H3K27, or H3R2), at least 10 blue and 10 orange peaks can be identified, supporting 10 well-positioned nucleosomes flanking the center CTCF site. The other datasets reveal 6–12 nucleosomes. The positions of these nucleosomes are in complete agreement with each other and with those seen for the mononucleosome mapping data (Figure 1B).

### Nucleosome Positioning around the Transcription Start Site and the Binding Sites of Other Transcription Factors

To compare the extent of nucleosome positioning by CTCF with that around the TSS, we applied GSA to the same mononucleosome mapping data [15] and the histone modification data [16] with the TSSs of actively transcribed genes as anchors. In agreement with previous findings [14],[15], there is a 200-bp-long nucleosome-free region around the TSS (indicated by a pronounced dip in the curves) and the +1 nucleosome is well-positioned, centered at ∼120 bp downstream of the TSS; in addition, two nucleosomes upstream of the TSS and four more nucleosomes downstream of the TSS are discernable (Figure S6A).

Among the histone modification datasets, the H3K4me3 dataset produced the strongest nucleosome-positioning signals, followed by H3K4me2. By combining H3K4me2 and H3K4me3 data, one can make out 5 positioned nucleosomes downstream of the TSS, with the first two apparent in the H3K4me3 curves and the last four discernable in the H3K4me2 curves (Figure S6B). Using TSS as anchors, the GSA curves of H3K27me1, H3K4me1 and H3K9me1 also show oscillatory behavior; however, the peaks are poorly formed, similar to those of H3K4me2 (Figure S6C). The GSA curves of other histone modification data do not show oscillatory behavior (figures not shown). The distance between the centers of neighboring nucleosomes measured in the TSS-anchored graphs agrees with that in CTCF-centered graphs. Collectively, these results indicate that there are 2 and 5 positioned nucleosomes upstream and downstream of TSS, respectively; however, the sharp contrast between Figure S6 with Figures 1B and 2 indicates that the positions of the nucleosomes around the TSS vary among different loci to a much greater extent than the positions of the nucleosomes flanking occupied CTCF sites.

We also investigated whether there were well-positioned nucleosomes flanking the binding sites of other transcription factors. The genome-wide maps of a number of transcription factors in living human cells have been produced using ChIP-chip or ChIP-Seq. Among them, the binding regions of STAT1, NRSF and p53 are highly enriched in the cognate motifs of these factors, which allowed us to determine the occupied sites by scanning the ChIP-chip or ChIP-Seq target regions with the motif matrices. We used these three sets of sites as anchors to produce aggregation plots with the mononucleosome mapping dataset [15] (Figure S7) and the histone modification datasets [16] (figures not shown). None of the graphs in Figure S7A/B/C show oscillatory behavior as in Figure 1B (the four graphs are drawn in the same scale), suggesting that these transcription factors do not possess the ability to position nucleosomes. The STAT1 and NRSF graphs indicate that the binding sites of these two factors have higher nucleosome occupancy than neighboring genomic positions, suggesting that their functions may be regulated by nucleosome positioning.

### The Histones Flanking Occupied CTCF Site Are Enriched in H2A.Z and 11 Histone Modifications

**Figure 3:**
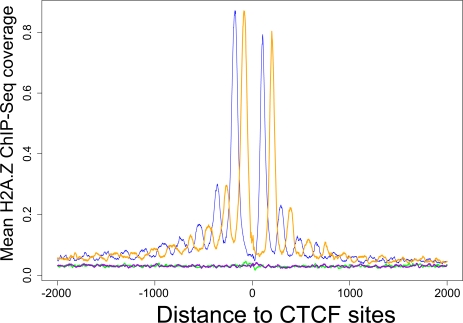
Nucleosomes flanking occupied CTCF sites are highly enriched in H2A.Z. The figure shows aggregated H2A.Z ChIP-Seq tag coverage on the plus and minus strands of occupied (blue and orange curves) or unoccupied (green and purple) CTCF sites. Note that the blue and orange curves are much higher than the green and purple curves.

We wanted to investigate whether some of the nucleosomes surrounding the occupied CTCF sites were enriched in H2A.Z or any of the histone modifications, in comparison with the nucleosome surrounding the unoccupied CTCF sites. We applied GSA to the ChIP-Seq dataset of H2A.Z with the occupied sites or the unoccupied CTCF sites as anchors, respectively, and obtained two sets of curves as shown in Figure 3. The green and purple curves are completely flat, again suggesting that nucleosomes are not well-positioned around unoccupied CTCF sites. Moreover, Figure 3 indicates the histones that flank the occupied CTCF sites are highly enriched in H2A.Z, especially the −1 and +1 nucleosomes, although the enrichment can be seen across a +/−2 kb region in Figure 3 (the blue and orange curves are cleanly above the green and purple curves).

**Figure 4:**
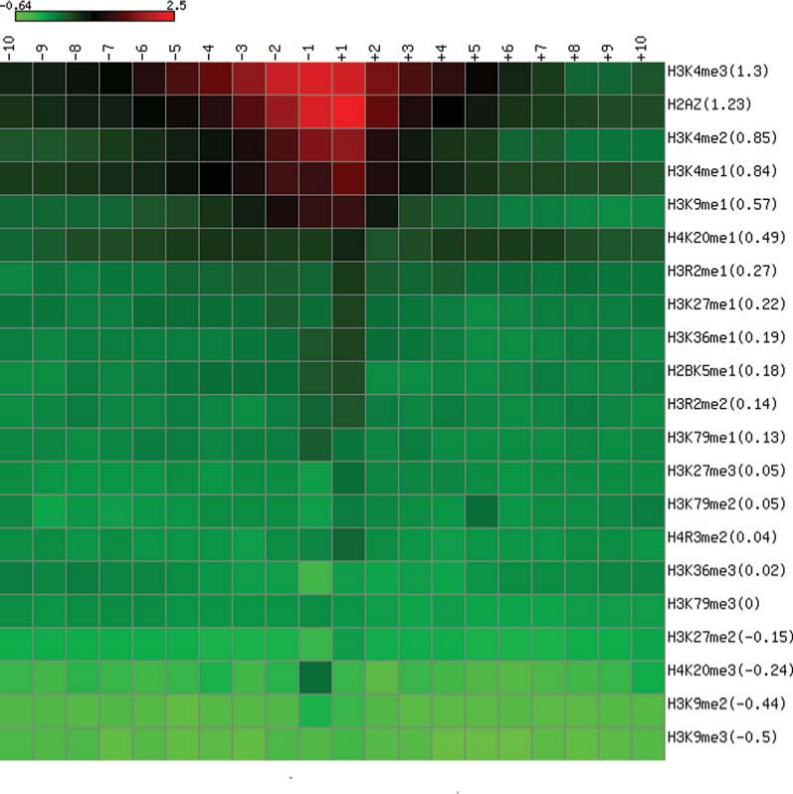
Enrichment of individual nucleosomes in H2A.Z or histone modifications. The columns index the nucleosomes flanking the anchoring CTCF site as in Figure 1A. Each row indicates one histone variant or modification, in descending order of overall enrichment. The numbers in parentheses indicate logarithm of average enrichment over 20 nucleosomes. Each cell shows log(enrichment) for a particular nucleosome, in a red to green color spectrum. Enrichment is defined as the ratio of the area under the curves for occupied and the area under the curves for unoccupied CTCF sites. The heatmap was created using matrix2png (http://www.bioinformatics.ubc.ca/pavlidis/lab/cgi-bin/matrix2png.cgi).

In order to account for the different sequencing depths among the datasets and the difference in nucleosome occupancy around occupied and unoccupied CTCF sites, we defined a histone variant or modification to be enriched if the ratio between the area under the curves anchored on occupied CTCF sites over the area under the curves anchored on unoccupied sites is higher than the ratio for mononucleosome mapping (1.07 as determined in the first section of Results), for the +/−2 kb region. By this criterion, subsets of nucleosomes flanking occupied CTCF sites are found to be enriched in H2A.Z and the following 11 histone modifications (in descending order of enrichment): H3K4me3, H3K4me2, H3K4me1, H3K9me1, H4K20me1, H3R2me1, H3K27me1, H3K36me1, H2BK5me1, H3R2me2, and H3K79me1. The other 9 histone modifications (H3K27me3, H3K79me2, H4R3me2, H3K36me3, H3K79me3, H3K27me2, H4K20me3, H3K9me2, and H3K9me3) are not enriched; nonetheless, most of them exhibit strong oscillatory patterns, with the best examples being H3K27me3 and H3K36me3 (Figure 2B). We applied the same criterion on consecutive 185 bp windows to determine whether individual nucleosomes are enriched in H2A.Z or the histone modifications. The resulting heatmap (Figure 4) reveals a large variation in how far different levels of enrichment spread from the CTCF anchors.

### The Footprints of CTCF on Genomic DNA against MNase or DNase I Cleavage

The aggregation graphs for more than half of the histone modifications in Figure 2 contain two extra prominent center peaks, which we suspect correspond to the 5′ and 3′ boundaries of the CTCF footprint. Take the H3K36me1 dataset as an example, because ChIP was performed with an antibody against H3K36me1 and not with an antibody against CTCF, we suggest that the blue peak resulted from the lack of digestion of the linker between the CTCF site and the +1 nucleosome; similarly, the orange peak resulted from the lack of digestion of the linker between the CTCF site and the −1 nucleosome. These two peaks coincide in position exactly with the only two peaks in the aggregation plot of the ChIP-Seq data of CTCF [16] with occupied CTCF sites as anchors (Figure 1C), the distance between which was determined to be 64 bp (see Methods). Note that sonication and not MNase digestion was used to generate the ChIP-Seq data of CTCF, thus there are no nucleosome peaks in Figure 1C. We do not observe the two center peaks for the mononucleosome mapping data (Figure 1B), nor for the H2A.Z data (Figure 3). Also, the occurrence of these peaks does not correlate with whether the nucleosomes are enriched in the particular histone modification. Thus, it is unclear whether the occurrence of these peaks merely reflects the experimental condition of the MNase digestion, or has biological significance.

Recently a genome-wide DNase I hypersensitivity map was produced on human CD4+ T cells with the DNase-Seq technology [20]. We applied GSA to this data with occupied CTCF sites as anchors, and the resulting pattern (Figure 1D) indicates that CTCF protects 30 bp of genomic DNA on the minus strand (the distance between the two inner orange peaks) and 42 bp on the plus strand (the distance between the two inner blue peaks) against DNase I digestion. The asymmetry between the protection lengths of the two strands is intriguing. We suggest that this is due to the closer contact of CTCF with the plus strand than with the minus strand. No crystal structure of a CTCF-DNA complex is available. Thus, we used the crystal structure of a six-zinc-finger protein with its cognate DNA [31] to model a CTCF-DNA complex. We computed the solvent accessible surface area of the two strands of the DNA in the crystal structure and determined the average area of one strand to be 23% higher than that of the other strand. Graphical display of the crystal structure indicates that the zinc-finger protein binds to the major groove of the DNA and that because the major groove has greater volume than the protein, the protein makes closer contact with one DNA strand than with the other stand. Lobanenkov and colleagues discovered that CTCF combined different subsets of its 11 zinc fingers when binding to divergent sites [24]–[26]. Such complexity can also result in asymmetric CTCF-DNA interaction.

The fine structure of the footprint shown in Figure 1D suggests that the non-zinc-finger portion of CTCF also contacts DNA and protects it from DNase I cleavage in a characteristic way. The two small peaks centered at −204 bp and +260 bp correspond to the linker between the −1 and −2 nucleosomes and the linker between the +1 and +2 nucleosomes, respectively.

### Sequence Conservation, Predicted Nucleosome-Forming Potential, and *in vitro* Reconstitution and Mapping of Nucleosomes for Genomic DNA around CTCF Sites

In order to test whether there is any evolutionary pressure on the primary sequences surrounding occupied CTCF sites, we obtained the phastCons scores for the sequences from the UCSC genome browser (http://genome.ucsc.edu) and plotted the average score at each position of the 4-kb window centered on occupied CTCF sites. In comparison, we also obtained the conservation for positions surrounding unoccupied CTCF sites. The two curves are shown in Figure 1E. It is apparent that conservation is only restricted to the center 15 bp of the CTCF binding motif with the highest information content (positions underlined in Figure S8). The positions 22–24 bp away from either side of the CTCF binding motif are even less conserved than the background, suggesting that these positions are not recognized by the CTCF in a sequence-specific manner.

Because the lack of sequence conservation at the mononucleotide level does not preclude these sequences from possessing intrinsic nucleosome-positioning ability, we applied a previously published computer algorithm that combines dinucleotide periodicity and a thermodynamic model to predict sterically allowed nucleosome placement [32]. We used the flavor of this algorithm trained with human data (see Methods). The algorithm predicts a nucleosome to occupy the CTCF sites that are occupied by CTCF in *vivo* (Figure S9), which corresponds to the linker region according to experimental data (orange and blue curves in Figure 1B and Figure 2). Thus, the algorithm predicts a nucleosome-positioning pattern that disagrees with the pattern experimentally measured around occupied CTCF sites.

**Figure 5:**
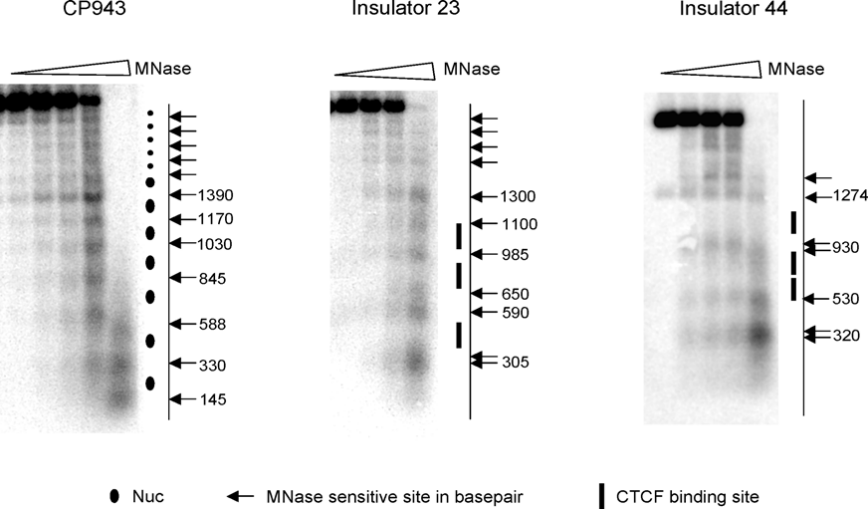
Analysis of *in vitro* nucleosome positioning surrounding CTCF binding sites. A 5S rDNA control fragment or insulator fragments that contain CTCF binding sites (insulator 23 and 24) were reconstituted into nucleosomal arrays with recombinant histone octamers. Reconstituted nucleosomal arrays were digested with increasing amounts of MNase and purified products were separated by agarose gel electrophoresis followed by Southern hybridization using radio-labeled oligonucleotides that anneal to one end of each array. Ovals denote positioned nucleosomes assembled on the head-to-tail repeats of the 5S rDNA nucleosome positioning sequences. Bars denote locations of CTCF binding sites. Note that CTCF sites are located between MNase cleavage sites and are likely to be occluded by nucleosomes.

To further test whether sequences that flank CTCF binding sites possess intrinsic nucleosome positioning signals, we reconstituted nucleosomes onto two different DNA fragments (Insulators 23 and 44) that each harbor three CTCF binding sites and were shown to function as insulators by enhancer blocking assays [33]. As a positive control, nucleosomes were also assembled onto a DNA fragment that contains 10 head-to-tail repeats of a 5S rDNA nucleosome positioning sequence (CP924). Nucleosomal arrays were digested with increasing amounts of micrococcal nuclease, and nucleosome positions were mapped by indirect end-labeling and Southern blot analysis. As shown in Figure 5, MNase analysis yielded a typical repeating pattern of cleavages and protections on the 5S repeat DNA, indicative of a positioned nucleosomal array. In contrast, nucleosomes assembled onto the DNA fragments that contain CTCF binding sites showed a much less regular pattern of MNase cleavages which is not consistent with a positioned nucleosomal array. Strikingly, each of the CTCF binding sites is located between MNase cleavages, indicating that these sites are bound by nucleosomes *in vitro*. Thus, these biochemical studies are in agreement with the predictions of the aforementioned computational algorithm (Figure S9), and they do not agree with the pattern of nucleosome positioning observed *in vivo* (Figure 1B and Figure 2).

### Measurement of Inter-Nucleosomal Distance, Lengths of Protected and Unprotected DNA, and CTCF Footprint Size

**Figure 6:**
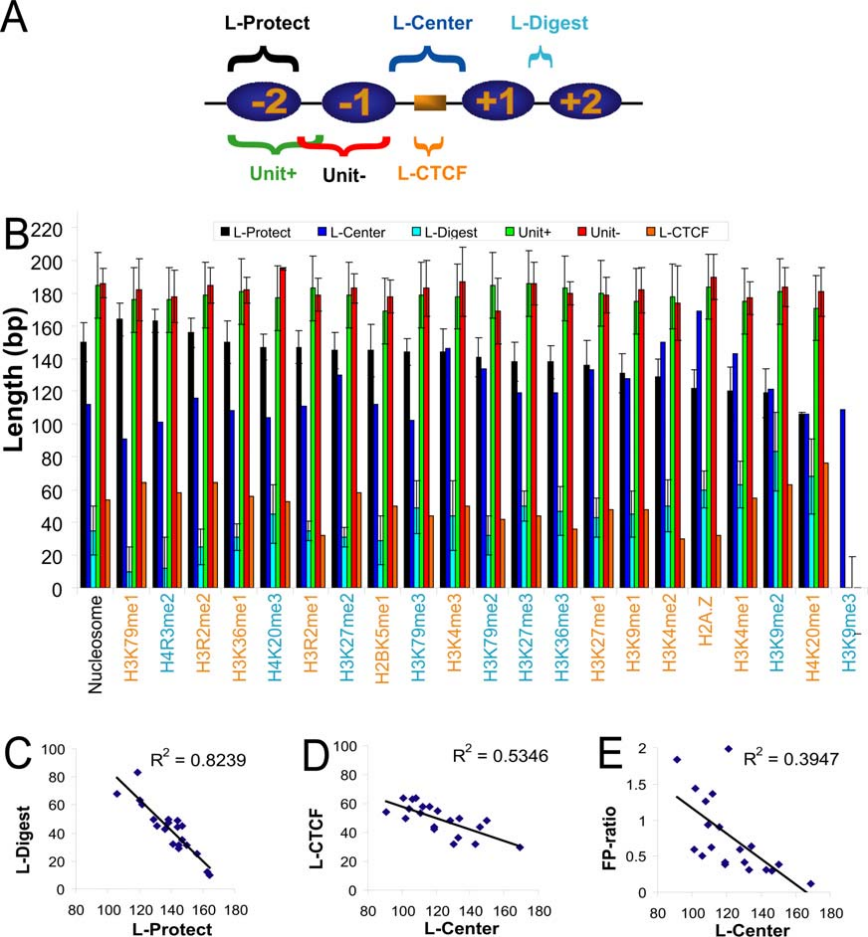
Great variation in DNA accessibility among H2A.Z and nucleosomes enriched in different histone modifications. Panel A: definition of terms. L-Protect, the length of DNA protected from MNase digestion; L-Center, the length of DNA digested by MNase between the −1 and +1 nucleosomes; L-Digest, the length of digested DNA between other neighboring nucleosomes; Unit+, distances between neighboring plus-strand peaks; Unit−, distances between neighboring minus-strand peaks; L-CTCF, length of CTCF micrococcal nuclease protection footprint. Panel B: bar graphs of distance measures. The datasets are arranged in the descending order of L-Protect, except for the mononucleosome mapping data (labeled nucleosome). Histone modifications colored in orange are enriched in occupied CTCF sites and the blue ones are not enriched; note the mixing of the datasets in the two colors. Panel C: negative correlation between L-Digest and L-Protect. Panel D: negative correlation between L-CTCF and L-Center. Panel E: negative correlation between FP-ratio and L-Center.

The well-positioned nucleosomes around CTCF sites provided an unprecedented opportunity to measure the distance between neighboring nucleosomes, the linker length, and the length of nucleosomal DNA protected against MNase cleavage. The distances between the +1 and −1 nucleosomes are increased due to the CTCF sites. We developed an algorithm to automatically determine the locations of the peaks in each GSA curve (see Methods). These peaks mark the boundaries of the well-positioned nucleosomes (Figures 1A/B and Figure S2). In order to relate the peak positions to the three aforementioned quantities, we defined six distances: L-CTCF, L-Center, L-Digest, Unit+, Unit− and L-Protect as illustrated in Figure 6A. Figure 6B plots these six distances for mononucleosome data, the 20 histone modifications and H2A.Z. For each dataset, the first two distances can be measured only once based on the two center nucleosomes while the last four distances can be measured multiple times for all but the H3K9me3 dataset and the standard deviations of these measurements are shown as error bars in Figure 6B. There is no statistically significant difference between distances measured on nucleosomes upstream and downstream of CTCF binding sites, or those between proximal and distal nucleosomes (data not shown).

It is apparent from Figure 6B that Unit+ and Unit− are largely invariant across the histone variants and histone modifications, consistent with the stable positions of the nucleosomes around the CTCF site. The length of nucleosomal DNA that are protected against MNase cleavage (L-Protect) ranges from 106 to 164 bp (139±15 bp) among the sets of nucleosomes enriched in different histone modifications or H2A.Z. The L-Protect determined with the mononucleosome mapping data [34] is 150 bp. The length of genomic DNA between two neighboring nucleosomes that is digested away by MNase (L-Digest) also varies greatly, from 10 to 83 bp (43±18 bp), with 35 bp for mononucleosome mapping data. L-Protect and L-Digest are strongly anti-correlated (R^2^ = 0.82 and P-value = 3e-8; Figure 6C), consistent with Unit+ and Unit− being largely invariant. These results may indicate that different histone modifications cause the ends of the nucleosomal DNA to wrap around the histone core with greatly varying extents of tightness. Tighter wrapping leads to lesser extent of MNase digestion and vice versa, without translational movement of the nucleosomes. Alternatively, different histone modifications may be associated with chromatin regions depleted in histone H2A/H2B dimers. Loss of dimers can be catalyzed by ATP-dependent remodeling enzymes that can be targeted to nucleosomes by histone modifications [35]. Loss of one H2A/H2B dimer will release ∼30 bp of nucleosomal DNA, and loss of both dimers yields an H3/H4 tetrasome particle that protects only ∼90 bp of DNA from MNase digestion [4].

Figure 6B indicates that H3K79me1 and H4R3me2 are the two histone modifications that lead to the best protection of nucleosomal DNA ends, while H3K9me2 and H4K20me1 are the two modifications that lead to the least protection. All 20 histone modifications are methylations, which do not change the net charges of the histones, thus the large variation among them is surprising. Moreover, different numbers of methyl groups on the same amino acid differ as much as the modifications on different amino acids. The histone modifications that correlate positively with gene expression level (H3K27me1, H3K9me1, H3K4me2, H3K4me1, H4K20me1, and to some extent H3K9me2 [16],[36]) correspond to the least protection against MNase, suggesting that increasing accessibility of the ends of nucleosomal DNA may be a mechanism for transcriptional activation. Nucleosomes enriched in H2A.Z have short L-Protect and long L-Digest, consistent with the effect of H2A.Z on transcriptional activation [10],[29].

The footprint of CTCF (L-CTCF) ranges from 32 to 64 bp among the datasets of different histone modifications (Figure 6B). With 11 fingers, CTCF can theoretically form direct atomic contact with 33 bp of DNA. The binding motif of CTCF has 15 positions with high information content (Figure S8), indicating that roughly five fingers contribute significantly to the binding specificity. Nonetheless, our results indicate that at least 32 bp of DNA is protected from MNase cleavage. L-CTCF strongly anti-correlates with the length of the MNase digested DNA between the −1 and +1 nucleosomes (L-Center, 118 bp for mononucleosome data), with R^2^ = 0.53 (P-value = 0.0002; Figure 6C). This suggests that when the DNA of these two nucleosomes is more accessible to MNase, the enzyme can cut closer to the CTCF site. Along this line of reasoning, we would also expect to see stronger MNase cleavage signal around the CTCF footprint for the histone modifications with greater L-Center. Because the nucleosomes flanking the CTCF site are enriched in different histone modifications to varying extents, we define the ratio between the average height of the CTCF footprint peaks and the average height of the +1 and −1 nucleosome peaks in Figure 2 as footprint-peak ratio (FP-ratio). Indeed, FP-ratio anti-correlates significantly with L-Center (R^2^ = 0.39 and P-value = 0.004; Figure 6E).

## Discussion

By integrating a large number of high-throughput sequencing and microarray datasets and performing aggregation analysis with transcription factor binding sites or the TSS as anchors, we discovered that there is an array of 20 nucleosomes flanking occupied CTCF sites genome-wide. These nucleosomes are so well positioned that remarkable oscillatory patterns were observed for 21 out of the 22 genome-wide datasets [16],[34]. Two case studies reported CTCF binding in the IGF2/H19 and DM1 loci, both of which suggested that the CTCF binding sites occurred in linker regions between nucleosomes [37],[38]. These are consistent with our findings in this study. We are unaware of other previous work on the genome-wide relationship between CTCF and nucleosomes. The TSS is the only genome-wide anchor for which well-positioned nucleosomes were reported, and only two nucleosomes upstream the TSS and five nucleosomes downstream of the TSS are well positioned. Here we show that 20 nucleosomes flanking CTCF sites exhibit much stronger oscillatory patterns, and hence are much better positioned than the nucleosomes around the TSS.

No well-positioned nucleosomes have ever been reported to flank transcription factor binding sites. Among the human transcription factors for which genome-wide binding data are available, the ChIP target regions for only three factors are highly enriched in their binding motifs (STAT1, NRSF and p53). We did not observe well-positioned nucleosomes around the occupied sites of any of these three factors. One complication is that the ChIP-Seq data of histone modifications were on CD4+ T cells while the binding site data were on other cell types. We must wait for future data to resolve this issue definitively, and to uncover whether well-positioned nucleosomes flank the binding sites of other transcriptional factors genome-wide.

There are four possible mechanisms for the well-positioned nucleosomes around CTCF sites: 1. CTCF binds to its sites first and then recruits chromatin remodeling factors to position neighboring nucleosomes; 2. CTCF binds to its sites first, which provides a strong anchor for the neighboring nucleosomes to line up by themselves; 3. Nucleosomes are well positioned in some regions of the genome due to DNA sequence features, and a CTCF site has co-evolved with the nucleosome positioning sequence features to exist in a lengthened linker region, which attracts the binding of CTCF; 4. Some genomic regions contain nucleosome-positioning sequence features leading to an array of regularly positioned nucleosomes which occlude a CTCF site, and CTCF binds to its site and repositions the nucleosomes to create a lengthened linker region. We argue that our results mostly support the second scenario for reasons as follows. Three lines of evidence suggest that the well positioned nucleosomes are unlikely caused predominantly by the intrinsic sequence features of the genomic DNA surrounding occupied CTCF sites: 1. There is a lack of conservation for the sequences that flank occupied CTCF sites, in sharp contrast with the strong conservation at the CTCF sites (Figure 1E); 2. A computational algorithm predicts a nucleosome to occupy sites that are occupied by CTCF *in vivo* (Figure S9); 3. We performed *in vitro* nucleosome reconstitution and mapping experiments on two insulators that each contains three CTCF sites. The results showed an irregular pattern of MNase cleavages, indicating the lack of a positioned nucleosomal array. Moreover, all six CTCF sites in these two insulators are in nucleosomal regions, consistent with the computational prediction and in contrast with the *in vivo* data. The binding of CTCF lengthens the linker region to 118 bp. Thus if the 20 nucleosomes form with regular intervals before CTCF binds, all of them will need to slide apart to accommodate the binding of CTCF, which seems unlikely. CTCF has not been reported to recruit chromatin remodeling factors. Thus we propose that the second scenario is most likely to be biologically relevant in general. Our hypothesis is consistent with the statistical positioning mechanism, which states that nucleosomes prefer not to occupy some regions of the genome due to sequence features such as homo-poly A/T or the eviction by regulatory proteins, but are well-positioned in the remaining regions of the genome due to structural constraints imposed by DNA packaging [39]. We hypothesize that the binding of CTCF acts as a roadblock for translational nucleosome movements and as a result the nucleosomes are packaged between the CTCF binding sites and the nearest nucleosome-free regions.

Nonetheless, our hypothesis does not preclude the possibility that in some loci other mechanisms cause well-positioned nucleosomes around CTCF sites. Indeed, Kanduri et al. reported that a subset of CTCF sites in the H19 locus was flanked by nucleosome positioning sequences and the authors argue that these sequences have evolved to ensure the constitutive availability of the CTCF binding sites [38]. Thus these results argue for the third scenario described above.

The nucleosomes flanking CTCF sites are enriched in H2A.Z and 11 histone modifications. Among these, H2A.Z and 8 histone modifications are also enriched in promoters and are positively correlated with the transcriptional levels of downstream genes [16]. The remaining three, H3K79me1, H3R2me1 and H3R2me2, are enriched to much less extents among the 11 modifications (Figure 4). The large overlap between the epigenetic features of nucleosomes in promoters and the nucleosomes around CTCF sites is surprising, given that CTCF is mostly known to bind to insulators, suggesting that CTCF may play an important role in regulating promoters.

The well-positioned nucleosomes around occupied CTCF sites allowed us to determine the length of the nucleosomal DNA protected against MNase digestion. Our results (Figure 6) indicate that there is great variation in the accessibility of nucleosomal DNA that corresponds to various histone methylations. It would be interesting to quantify the amounts of variation for modifications that affect net charges of the histones, once the data becomes available. The histone modifications that correspond to greater DNA accessibilities and H2A.Z, which also corresponds to great DNA accessibility, are highly enriched in promoters of expressed genes. Collectively, these results suggest that one of the mechanisms by which histone modifications regulate gene expression can be by modulating accessibility to the genomic DNA. In light of the recent findings on histone turnover [40],[41], it is tempting to suggest that accessible DNA would facilitate rapid histone turnover and/or rapid turnover results in accessible DNA. In particular, rapid histone turnover was observed in chromatin boundaries and suggested to help delimit the spread of chromosome states [40],[41]. Because the primary function of CTCF is to bind to insulators, which are the most well understood boundary elements, we suggest that those CTCF sites flanked by nucleosomes with highly accessible DNA can prevent the lateral spreading of chromosome states.

Figure 6 also suggests that regions around occupied CTCF sites are of heterogeneous composition: subsets of them are enriched in different histone modifications, therefore producing different L-Digest measurements. Indeed, hierarchical clustering of the regions surrounding all occupied CTCF sites based on the ChIP-Seq signal levels of histone modification, H2A.Z and RNA polymerase II (Figure S10) confirms that these genomic regions have diverse patterns of epigenetic marks. It would be interesting to investigate whether some of these patterns are correlated with the insulator function, and if so, which ones are. CTCF has also been reported to possess activating and repressing functions and it is possible that some epigenetic patterns correspond to these functions. Figure S10 further indicates that all the nucleosomes surrounding occupied CTCF sites are covered by H2A.Z and/or some of the histone modifications investigated in this study. Because well-positioned nucleosomes are observed for all but one histone modification datasets (Figure 2), we conclude that this is a universal feature of CTCF, regardless of the underlying biological function (insulation, activation, repression or others) of the particular locus.

Because Unit+ and Unit− are on average 185 bp and largely invariant, we can deduce that the length of human linker DNA is 38 bp given that 147 bp of DNA is observed in the crystal structure of nucleosomes [4]. This linker length is somewhat shorter than the previous estimate of 70 bp in higher eukaryotes [6]. Because our analysis included data on all nucleosomes, nucleosomes with H2A.Z or one of 20 histone modifications, we believe that 38 bp is a robust estimate. Furthermore, this is unlikely to be specific to only the nucleosomes flanking occupied CTCF sites, because the well-positioned nucleosomes around the TSS have similar intervals as the nucleosomes flanking CTCF sites.

In summary, we discovered that occupied CTCF binding sites in the human genome are flanked by 20 well-positioned nucleosomes. These nucleosomes are enriched in H2A.Z and 11 histone modifications, forming complex epigenetic patterns. Nucleosomes enriched in different histone modifications have diverse but compensating lengths of DNA that are protected from or digested by MNase. The binding of CTCF extends the linker to 118 bp and the CTCF footprint is smaller if the DNA of neighboring nucleosomes is more accessible. These results provide insights to the interplay between chromatin structure and CTCF function.

## Materials and Methods

### Data Source for Computational Analysis

The genomic coordinates of mapped sequence tags for the mononucleosome mapping datasets [34] and for ChIP-Seq datasets [16] were kindly provided to us by Schones and Zhao. As in those two studies, only those sequenced reads that map uniquely to the human genome (hg18) were used for all analyses in our study.

Kim *et al.* annotated CTCF binding sites based on ChIP-chip data with an antibody against CTCF on IMR90 cells [17]. CTCF binding has been reported to be largely ubiquitous across multiple cell types [17],[33]. Thus we took the subset of the CTCF sites annotated by Kim et al. within ChIP-chip target regions that are also overlap with CTCF ChIP-Seq data in CD4+ T cells [16], and defined them as the *occupied* CTCF sites, used throughout this study. A subset of CTCF sites annotated by Kim et al. using the CTCF binding matrix to scan genomic regions [17], which are outside the target regions of both the aforementioned ChIP-chip and ChIP-Seq datasets but within the scopes of these two experiments, were defined as *unoccupied* sites. A small number of these sites may be occupied by CTCF in cell types that have not been studied, but this does not affect our conclusions, because the vast majority of data on which we based our analysis was generated on CD4+ T cells. In total, we define 6432 occupied sites and equal number of unoccupied sites and the lists are available as Table S1.

Genome-wide DNase-Seq data was obtained from [20]. Transcription start sites for known genes were downloaded from the UCSC genome browser (http://genome.ucsc.edu), and partitioned according to expression levels measured by Su *et al.* [42]. Occupied STAT1 binding sites were determined by scanning STAT1 ChIP-Seq target regions with TRANSFAC matrix M00223 [43]. Occupied NRSF binding sites were annotated by Johnson *et al.* [19]. Occupied p53 binding sites were annotated by applying p53-PET model in ChIP-PET (Paired End di-Tag) sequences with at least 3 tags [18].

### Genomic Signal Aggregation (GSA)

Many types of genomic data including ChIP-Seq, ChIP-chip, MNase-Seq or DNase-Seq can be represented as a set of genomic positions, each associated with a score. For example, the ChIP-chip raw data is constituted of a set of short oligonucleotide probes each associated with a hybridization intensity score. These probes are mapped onto the genome, which assigns their scores to all the corresponding genomic positions. ChIP-Seq, MNase-Seq and DNase-Seq yield sequence reads. After all the reads are mapped to the genome, each genomic position can be assigned a score which corresponds to how many reads that cover the position.

One type of highly informative analysis to perform on such genomic data is to aggregate over a set of genomic anchors at base-pair resolution. The analysis yields a continuous curve, with the average score for the genomic position at a particular distance away from the anchor plotted against the distance. In this study, we use two types of anchors, transcriptional start sites or the 5′-ends of transcription factor binding sites. We developed the genomic signal aggregation (GSA) algorithm for performing aggregation analysis (Figure S1). Specifically, each genomic position is assigned to the nearest anchor and classified as upstream, on-anchor or downstream, and the scores for all the positions at a specified distance from the anchor are averaged. In order to account for the scenario that a position at a certain distance to the anchor may correspond to more genomic locations than another position, due to the uneven distribution of the anchors in the genome, the sums of scores are divided by the numbers of contributing genomic locations instead of by the number of anchors. An earlier version of this algorithm was applied to a large number of ChIP-chip data generated by the ENCODE consortium [44]. We have built a freely accessible web server for GSA (http://zlab.bu.edu/GSA).

ChIP-Seq, MNase-Seq or DNase-Seq datasets are composed of sequence reads, which could map to either the same or the opposite strand of the nearest anchor. We separately performed GSA on the reads that map to the two strands, as indicated in Figure S2, in order to reveal fine details of the data. This leads to the two sets of curves throughout this study (e.g., the orange and blue curves in Figure 1). We assigned the count of a sequence tag to all the positions of the sequence reads (24 bp or longer) instead of only to the 5′-end position, in order to smooth the curves. As a result, the peaks of the curves are not at the boundaries of the nucleosome, but are 12 bp inside the boundaries as illustrated in Figure S2 and all the GSA curves throughout this paper. Figure 6 defines distances between the exact nucleosome boundaries by factoring in the 12 bp. For the DNase-Seq data, we only used the 5′-end position for aggregation because there was sufficient number of tags (12 M), thus there is no such 12-bp shift.

The y-axis of the GSA curve for a ChIP-Seq, MNase-Seq or DNase-Seq dataset indicates the average number of sequence reads that are mapped to a particular distance to an anchor, with the average taken over all anchors. Thus the height of a GSA curve depends on the sequencing depth. For example, 154.6 M reads were used to generate Figure 1B (all mono-nucleosomes) and 10.1 M reads were used to generate the H3K4me3 panel in Figure 2A, which directly accounts for the difference in the y-axis spans of these two figures. Figure S3 includes the remake of Figure 1B with 20% or 5% randomly sampled reads, and it is apparent that the heights of the curves in Figure S3 decrease proportionally when compared with those in Figure 1B. See the second paragraph of Results for discussion on how to compare GSA curves.

The result of GSA analysis on nucleosome positioning is affected by the experimental method used to fragment the chromatin samples. For most of the results discussed in this article, we used the datasets prepared with MNase digestion: the all mononucleosome mapping dataset with 154.6 M sequence reads [15], and the 20 histone modification ChIP-Seq datasets in [16]. To contrast MNase digestion with sonication, we also produced the GSA plots on another dataset in [15], generated with ChIP of H3 followed by sonication to 200–300 bp long DNA fragments (Figure S11). Only 12 well-positioned nucleosomes are discernable around occupied CTCF sites (Figure S11A), in contrast with 20 nucleosomes seen with the MNase digestion dataset (Figure 1B). Consistent with the finding reported previously [15], only 3 well-positioned nucleosomes are discernable around the TSSs of expressed genes (Figure S11B), in contrast with 7 nucleosomes seen with the MNase digestion dataset (Figure S6A).

### Peak Annotation and Distance Measurement

We wrote a PERL program for calling peaks in GSA curves. It searches for the local maxima compared with their flanking intervals, which were set to 70 bp for detecting nucleosome peaks and 15 bp for detecting CTCF footprints. Each inter-nucleosome distance was measured as the mean of the distance between neighboring plus-strand peaks and the distance between the corresponding minus-strand peaks. The counts of well-positioned nucleosomes start at the center of CTCF binding sites and continue in both directions until the variation between inter-nucleosome distances exceeds 40 bp. Positions of peaks and counts of well-positioned nucleosomes were visually inspected and minor defects due to imperfectly formed peaks were corrected.

### Prediction of Nucleosome-Positioning Potential

The 3 kb sequences flanking CTCF binding sites were downloaded from the UCSC genome browser (hg18). The sequences were fed to the program by Segal et al. [32] for predicting nucleosome occupation probability, with the human nucleosome model (both downloaded from http://genie.weizmann.ac.il/pubs/nucleosomes06/). The predicted per-base-pair probability values were sampled every 50 bp and then aggregated using GSA, with the occupied and unoccupied CTCF binding sites as anchors, separately.

#### Reconstitution of Nucleosomes

Oligonucleotides (Integrated DNA Technologies, Inc., Coralville, IA) were 5′ end labeled with ^32^P using γ-^32^P -ATP and T4 polynucleotide kinase (New England Biolabs, Inc., Beverly, MA). Plasmid CP943 (p2085S-G5E4) and plasmids containing the Insulator sequences were prepared by alkaline lysis method. Plasmid CP943 was digested with *Nde*I+*Cla*I to release the array of 5S rDNA nucleosome positioning elements (∼2.3 kb). Likewise, plasmids containing Insulator 23 (pTVIns023) and Insulator 44 (pTVIns044) were digested with *Not*I/*Kpn*I and *Bam*HI/*Kpn*I respectively to release the1.2–1.6 kb insulator fragments. Recombinant Xenopus histones and octamer were purified and nucleosomal arrays were reconstituted as described earlier [45], using one octamer per 200 base-pairs of DNA, i.e. R = 1.0.

#### MNase-Southern Assay

Reconstituted nucleosomal arrays were subjected to MNase digestion followed by Southern hybridization to assess the nucleosomal occupancy. To this end, 1 µg DNA equivalent of each of the reconstituted chromatin was digested with various amounts of MNase (Worthington), serially diluted from 15 units. Reactions were incubated at 25°C in a reaction buffer containing 10 mM Tris-HCl pH 8.0, 2 mM MgCl_2_, 75 mM NaCl and 0.3 mM CaCl_2_. After 15 seconds, reactions were stopped by addition of 2.5 mM EDTA, 2.5 mM EGTA, 1% SDS and 1 mg/ml Proteinase K. Reactions were incubated further for 15 minutes and then extracted twice with phenol-chloroform. Purified products were then resolved by 1.5% agarose gel electrophoresis, followed by Southern Hybridization using 32P-labelled oligonucleotide probes that anneal adjacent to the *Not*I site of Insulator 23 and the *Kpn*I site of Insulator 24 fragments. The sequences of these to insulators are provided in Table S2.

## Supporting Information

Figure S1 Illustration of anchor assignment and signal averaging in GSA. Black arrows and dashes represent anchors and sequence tags, respectively. Rectangles represent a genomic region interrogated by the experiment (repetitive regions are often not interrogated). One sequence tag (in gray) is mapped to the mid-point between anchors 1 and 2 and hence distributes its contribution equally to these two anchors. After all positions in the genomic region are attributed to the nearest anchor, they are aligned in a strand-specific way (plus and minus strands with respect to the anchor are indicated with the + and − signs), and the score for each position indicates the average number of sequence tags that cover that position.
(0.66 MB EPS)

Figure S2 Illustrations on the source of doublet peaks in aggregation plots of MNase-Seq and ChIP-Seq tags. Because ChIP-Seq tags were mapped to specific strands of the reference human genome, they can be separated by whether they reside on the same or the opposite strand of the anchor. Because mono-nucleosomal DNA is on average 147-bp long, and the sequence tags are on average 24 bp long, the distance between the plus- and minus-strand peaks is ∼123 bp.
(0.63 MB EPS)

Figure S3 Impact of sequencing depth on the aggregation analysis. A. 20% and B. 5% randomly sampled sequence reads were used to make the aggregation graph as in Figure 1B.
(2.86 MB EPS)

Figure S4 Nucleosome occupancy around occupied CTCF sites, in comparison with unoccupied CTCF sites. The ratio of the sum of area under the blue and orange curves (anchored on occupied CTCF sites) over the sum of area under the green and purple curves (anchored on unoccupied CTCF sites) in Figure 1B is shown for each nucleosome.
(0.67 MB EPS)

Figure S5 Aggregation over subsets of occupied CTCF sites. For purple and green curves, occupied CTCF sites that are 2–5 kb away from the nearest occupied CTCF sites were used as anchors for aggregation. For blue and orange curves, occupied CTCF sites that are more than 500 kb away from the nearest occupied CTCF sites were used as anchors for aggregation. Purple and orange: minus strand; green and blue: plus strand. Occupied CTCF sites less than 2 kb away from the nearest occupied sites are excluded from this analysis because the aggregation is performed over a 2-kb distance.
(1.67 MB EPS)

Figure S6 TSS positions nucleosomes less strongly than CTCF. A. Average sequence coverage corresponding to all nucleosomes, anchored on the transcription start sites of expressed genes. Seven well-positioned nucleosomes are seen, two upstream and five downstream. B. Aggregation of the ChIP-Seq data on H3K4me3 (blue and orange curves for plus- and minus-strand tags, respectively) and H3K4me2 (green and purple), plotted against the transcription start sites of expressed genes. Approximate positions of the plus and minus strand peaks are indicated with black vertical lines. C. Aggregation of the ChIP-Seq data on H3K27me1, H3K4me1 and H3K9me1 around the transcription start sites of expressed genes. Rough peaks can be made out from these plots. The plots for other histone modifications do not show oscillatory behavior.
(3.06 MB EPS)

Figure S7 Aggregated sequence tag coverage around occupied STAT1, NRSF and p53 binding sites in the human genome. The A/B/C graphs were produced using the MNase-Seq data and drawn in the same scale as Figure 1B. Unlike Figure 1B, none of these plots exhibit oscillatory behavior, indicating that there are no well-positioned nucleosomes. The D/E graphs were produced using the ChIP-Seq data of STAT1 and NRSF, respectively. These graphs show strong double peaks, corresponding to the boundaries of the footprints of these two transcription factors on genomic DNA. The data of p53 did not have enough tags for producing a GSA graph.
(4.09 MB EPS)

Figure S8 Sequence logo of CTCF. The logo was produced with all occupied sites, with the WebLogo server (http://weblogo.berkeley.edu). The 15 positions with high information content are underlined. All the GSA plots in this paper use the first position (labeled 0) as the x-coordinate origin.
(0.57 MB EPS)

Figure S9 Aggregated nucleosome occupancy probability around CTCF sites. The Y-axis indicates probability of a base pair being covered by a nucleosome from the program by Segal et al. [32]. The green curve is for occupied CTCF sites and purple for unoccupied sites. The fluctuation can be attributed to the distance constraints hard-coded in the algorithm.
(0.89 MB EPS)

Figure S10 Two-way hierarchical clustering of occupied CTCF sites by their histone modification patterns in flanking nucleosomes. Each row represents a CTCF site and each cell represents logarithmic normalized count of ChIP-Seq tags that correspond to a histone modification, H2A.Z, or Pol II, within 150 bp. High and low counts are represented by orange and blue colors, respectively.
(0.56 MB PDF)

Figure S11 Figure S11. Aggregation figures generated with the H3 ChIP-Seq sonication data. The dataset from CTCF sites (A) TSSs of expressed genes (B) were used as anchors.
(1.64 MB EPS)

Table S1 Lists of hg18 coordinates for occupied and unoccupied CTCF sites.
(0.34 MB TXT)

Table S2 Sequences of Insulators 23 and 44, with CTCF sites highlighted in yellow.
(0.09 MB DOC)

## References

[1] Levitsky VG, Podkolodnaya OA, Kolchanov NA, Podkolodny NL (2001). Nucleosome formation potential of eukaryotic DNA: calculation and promoters analysis.. Bioinformatics.

[2] Lee CK, Shibata Y, Rao B, Strahl BD, Lieb JD (2004). Evidence for nucleosome depletion at active regulatory regions genome-wide.. Nat Genet.

[3] Sekinger EA, Moqtaderi Z, Struhl K (2005). Intrinsic histone-DNA interactions and low nucleosome density are important for preferential accessibility of promoter regions in yeast.. Mol Cell.

[4] Harp JM, Hanson BL, Timm DE, Bunick GJ (2000). Asymmetries in the nucleosome core particle at 2.5 A resolution.. Acta Crystallogr D Biol Crystallogr.

[5] Oliver SG, Broda P, Sims PFG, Society for General Microbiology. Symposium (50th: 1993: UMIST) (1993). The eukaryotic genome: organisation and regulation.

[6] Williams SP, Athey BD, Muglia LJ, Schappe RS, Gough AH (1986). Chromatin Fibers Are Left-Handed Double Helices with Diameter and Mass Per Unit Length That Depend on Linker Length.. Biophysical Journal.

[7] Guillemette B, Bataille AR, Gevry N, Adam M, Blanchette M (2005). Variant histone H2A.Z is globally localized to the promoters of inactive yeast genes and regulates nucleosome positioning.. PLoS Biol.

[8] Li B, Pattenden SG, Lee D, Gutierrez J, Chen J (2005). Preferential occupancy of histone variant H2AZ at inactive promoters influences local histone modifications and chromatin remodeling.. Proc Natl Acad Sci U S A.

[9] Raisner RM, Hartley PD, Meneghini MD, Bao MZ, Liu CL (2005). Histone variant H2A.Z marks the 5′ ends of both active and inactive genes in euchromatin.. Cell.

[10] Zhang H, Roberts DN, Cairns BR (2005). Genome-wide dynamics of Htz1, a histone H2A variant that poises repressed/basal promoters for activation through histone loss.. Cell.

[11] Yuan GC, Liu YJ, Dion MF, Slack MD, Wu LF (2005). Genome-scale identification of nucleosome positions in S. cerevisiae.. Science.

[12] Lee W, Tillo D, Bray N, Morse RH, Davis RW (2007). A high-resolution atlas of nucleosome occupancy in yeast.. Nat Genet.

[13] Albert I, Mavrich TN, Tomsho LP, Qi J, Zanton SJ (2007). Translational and rotational settings of H2A.Z nucleosomes across the Saccharomyces cerevisiae genome.. Nature.

[14] Ozsolak F, Song JS, Liu XS, Fisher DE (2007). High-throughput mapping of the chromatin structure of human promoters.. Nat Biotechnol.

[15] Schones DE, Cui K, Cuddapah S, Roh TY, Barski A (2008). Dynamic regulation of nucleosome positioning in the human genome.. Cell.

[16] Barski A, Cuddapah S, Cui K, Roh TY, Schones DE (2007). High-resolution profiling of histone methylations in the human genome.. Cell.

[17] Kim TH, Abdullaev ZK, Smith AD, Ching KA, Loukinov DI (2007). Analysis of the vertebrate insulator protein CTCF-binding sites in the human genome.. Cell.

[18] Wei CL, Wu Q, Vega VB, Chiu KP, Ng P (2006). A global map of p53 transcription-factor binding sites in the human genome.. Cell.

[19] Johnson DS, Mortazavi A, Myers RM, Wold B (2007). Genome-wide mapping of in vivo protein-DNA interactions.. Science.

[20] Boyle AP, Davis S, Shulha HP, Meltzer P, Margulies EH (2007). High-resolution mapping and characterization of open chromatin across the genome.. Cell In press.

[21] Lee JT (2003). Molecular links between X-inactivation and autosomal imprinting: X-inactivation as a driving force for the evolution of imprinting?. Curr Biol.

[22] Burcin M, Arnold R, Lutz M, Kaiser B, Runge D (1997). Negative protein 1, which is required for function of the chicken lysozyme gene silencer in conjunction with hormone receptors, is identical to the multivalent zinc finger repressor CTCF.. Mol Cell Biol.

[23] Klenova EM, Nicolas RH, Paterson HF, Carne AF, Heath CM (1993). CTCF, a conserved nuclear factor required for optimal transcriptional activity of the chicken c-myc gene, is an 11-Zn-finger protein differentially expressed in multiple forms.. Mol Cell Biol.

[24] Ohlsson R, Renkawitz R, Lobanenkov V (2001). CTCF is a uniquely versatile transcription regulator linked to epigenetics and disease.. Trends Genet.

[25] Renda M, Baglivo I, Burgess-Beusse B, Esposito S, Fattorusso R (2007). Critical DNA binding interactions of the insulator protein CTCF: a small number of zinc fingers mediate strong binding, and a single finger-DNA interaction controls binding at imprinted loci.. J Biol Chem.

[26] Filippova GN, Fagerlie S, Klenova EM, Myers C, Dehner Y (1996). An exceptionally conserved transcriptional repressor, CTCF, employs different combinations of zinc fingers to bind diverged promoter sequences of avian and mammalian c-myc oncogenes.. Mol Cell Biol.

[27] Bulger M, Groudine M (1999). Looping versus linking: toward a model for long-distance gene activation.. Genes Dev.

[28] Kurukuti S, Tiwari VK, Tavoosidana G, Pugacheva E, Murrell A (2006). CTCF binding at the H19 imprinting control region mediates maternally inherited higher-order chromatin conformation to restrict enhancer access to Igf2.. Proc Natl Acad Sci U S A.

[29] Lobanenkov VV, Nicolas RH, Adler VV, Paterson H, Klenova EM (1990). A novel sequence-specific DNA binding protein which interacts with three regularly spaced direct repeats of the CCCTC-motif in the 5′-flanking sequence of the chicken c-myc gene.. Oncogene.

[30] Yusufzai TM, Tagami H, Nakatani Y, Felsenfeld G (2004). CTCF tethers an insulator to subnuclear sites, suggesting shared insulator mechanisms across species.. Mol Cell.

[31] Segal DJ, Crotty JW, Bhakta MS, Barbas CF, Horton NC (2006). Structure of Aart, a designed six-finger zinc finger peptide, bound to DNA.. J Mol Biol.

[32] Segal E, Fondufe-Mittendorf Y, Chen L, Thastrom A, Field Y (2006). A genomic code for nucleosome positioning.. Nature.

[33] Xi H, Shulha HP, Lin JM, Vales TR, Fu Y (2007). Identification and characterization of cell type-specific and ubiquitous chromatin regulatory structures in the human genome.. PLoS Genet.

[34] Schones DE, Smith AD, Zhang MQ (2007). Statistical significance of cis-regulatory modules.. BMC Bioinformatics.

[35] Vicent GP, Nacht AS, Smith CL, Peterson CL, Dimitrov S (2004). DNA instructed displacement of histones H2A and H2B at an inducible promoter.. Mol Cell.

[36] Heintzman ND, Stuart RK, Hon G, Fu YT, Ching CW (2007). Distinct and predictive chromatin signatures of transcriptional promoters and enhancers in the human genome.. Nature Genetics.

[37] Filippova GN, Thienes CP, Penn BH, Cho DH, Hu YJ (2001). CTCF-binding sites flank CTG/CAG repeats and form a methylation-sensitive insulator at the DM1 locus.. Nat Genet.

[38] Kanduri M, Kanduri C, Mariano P, Vostrov AA, Quitschke W (2002). Multiple nucleosome positioning sites regulate the CTCF-mediated insulator function of the H19 imprinting control region.. Mol Cell Biol.

[39] Kornberg RD, Stryer L (1988). Statistical distributions of nucleosomes: nonrandom locations by a stochastic mechanism.. Nucleic Acids Res.

[40] Dion MF, Kaplan T, Kim M, Buratowski S, Friedman N (2007). Dynamics of replication-independent histone turnover in budding yeast.. Science.

[41] Mito Y, Henikoff JG, Henikoff S (2007). Histone replacement marks the boundaries of cis-regulatory domains.. Science.

[42] Su AI, Wiltshire T, Batalov S, Lapp H, Ching KA (2004). A gene atlas of the mouse and human protein-encoding transcriptomes.. Proc Natl Acad Sci U S A.

[43] Wingender E, Chen X, Hehl R, Karas H, Liebich I (2000). TRANSFAC: an integrated system for gene expression regulation.. Nucleic Acids Res.

[44] Birney E, Stamatoyannopoulos JA, Dutta A, Guigo R, Gingeras TR (2007). Identification and analysis of functional elements in 1% of the human genome by the ENCODE pilot project.. Nature.

[45] Luger K, Rechsteiner TJ, Richmond TJ (1999). Expression and purification of recombinant histones and nucleosome reconstitution.. Methods Mol Biol.

