# Supplementary figures and images for: The Insulator Binding Protein CTCF Positions 20 Nucleosomes around Its Binding Sites across the Human Genome
Source: PLoS Genet. 2008 Jul 25;4(7):e1000138. doi: 10.1371/journal.pgen.1000138 (PMC2453330; doi:10.1371/journal.pgen.1000138)

# Hierarchical Clustering UPGMA Correlation Average

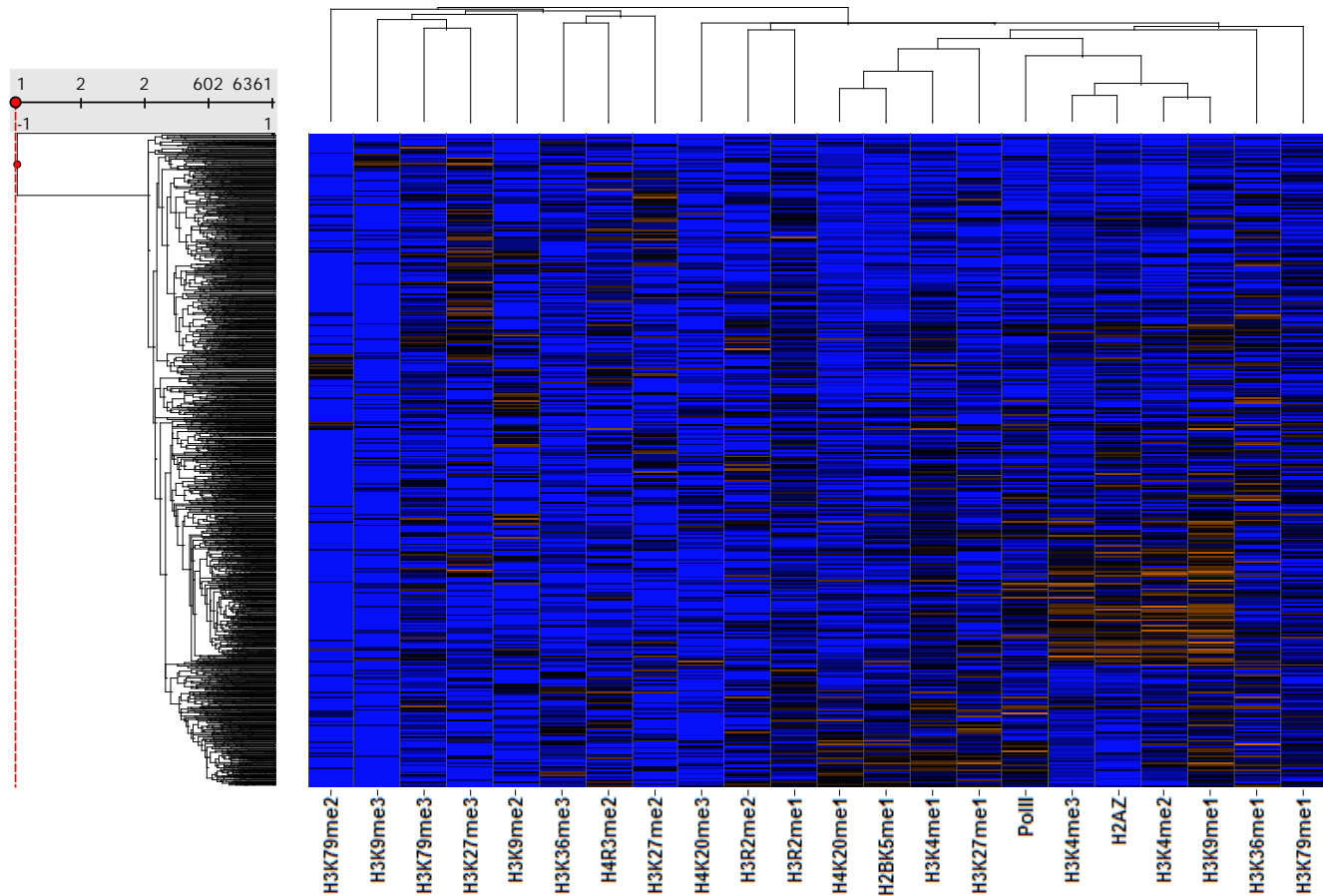

Supplement: Figure S10 — Two-way hierarchical clustering of occupied CTCF sites by their histone modification patterns in flanking nucleosomes. Each row represents a CTCF site and each cell represents logarithmic normalized count of ChIP-Seq tags that correspond to a histone modification, H2A.Z, or Pol II, within 150 bp. High and low counts are represented by orange and blue colors, respectively. (0.56 MB PDF) [file pgen.1000138.s010.pdf]
