# Supplementary material for: The Insulator Binding Protein CTCF Positions 20 Nucleosomes around Its Binding Sites across the Human Genome
Source: PLoS Genet. 2008 Jul 25;4(7):e1000138. doi: 10.1371/journal.pgen.1000138 (PMC2453330; doi:10.1371/journal.pgen.1000138)
Supplement: Table S2 — Sequences of Insulators 23 and 44, with CTCF sites highlighted in yellow. (0.09 MB DOC) [file pgen.1000138.s013.doc]

**Insulator 23 sequence (CTCF sites are highlighted in yellow):**

ggtaccctgtttccttatctgacctgctttaactgggtaagcttatgaaaagtcttgtgtagaagagaaagggataacagcctgtgctaaatgaggaagctgccttgcctgttcctgctcagtggggtttctggctatactacatcaactgagtcagtgcatcttgactagttcccacacctttctctgaacagagagagtaaagggctcaataagaaaaatacagtttatggtctgtacgttgtattacacgtcatcccttactttctaaaaggcatcttcactgagaaagacatggatttctaaccttacaatctctacaaagttcagaatacataataatcttgaattatgattaactgttagttttgaccaggtcttctggcagacaggtcacatgtgttagtatcacttattcctcaagtgttgatgttagtgtcagcatattaccgatgttccacaaacatttcctgaatgactgttaaacttcctacacattaacgagcctctgcatttttttccagcttccatctatgatttaagtaaactctagttttccacttcttcatattctctctctagatctcaattattgcagtaccactgtccaagggcagaggaggttagctgggcccaggcggagtcaattctctactccccaccctgtgggtgtgttcagccttgtgagccagcatcaggcttgagcacagcagtgctgagtcatgctgagtcatgctgaggcttagggtgtgtggccagatgttttcagctgtgagtgatcagtgctatctgggtctctaggaggaagtccacagggaaggtgaaaagaaaataagtttgctccctgaagaaaacattacttacaccagcattacaatgaaaaggggaccctgccttgctgtgtgacataacctagaatattttattttctagttaaaaattacttctcatgatctttattagaagttataacaaatcattttttagaaatgtaaagtttatttagactcatacctagaagcaacagaatcacacattttagtgtacacacacacacacacacgcacacacacacacatgacttttgttcataaactatgcaattgttaatgaaatgctatttggaatgggcttgaattgcacgggccccccctcgaggtcgacggtatcgatagagatgtgcctggaaccactcctgatgccatggctagagtggcccccctgacttctgcccggggtgttgacctcgcctcacctttcacagaccagaccggcacaggctttgagctcattataaccacactccttgtctcgtgtctgcctcactttcttggtttcatctttggtttctcaggtcatacttatccatttggaattggaagaattctattttcagggttatcaaaaccagggagaggtttgagtcaaggagagtccttgggagaaaaagagagaatcgagactacagaaggccagagctgggccttatcatcatcccccaggccccctcaagtgcagccgaggagcgggcggtaggggtgggggcactcaagctctgctcaagatcacatgcccatgaatccaacccctgcttagcaagacggaagaaccggggaggggagagggagaaagttgctccattgtagctactggagaaatcagaggctgtggggccacagagggaatggaaaatgtctctcttcttttcttgatctctggccttcccaggagcagagatagcagcccaatcccttagacgccagggtgatgccggatttggggaccctggaccattttagattctgctggcttagagaggcagttctgggattcctgaggccactccttgtgcaacagtctcagttttgccagaaataacctgggggacttgaggggagcgggagcccattgcgtcatagttgctaggcaacccccagcctgaaggagcgcatcgtcccccgttgccctccggtggccgctctcgcatgctgcaggctctgtcccgactgaggaatagcagcactgccacctggtggatccgcggcgctcagagagcccattcaagcgcatccagttcctcccgcggagaccaagcccggtcaggcaagttccgtgttctgcagctctgggcagctcagagcttctgaggctggggatccctcagctccacgttagcggtttcctccggagctggtgctgctgctttcttctctttttccaattttgctccccaatgtgctgaactcctatgaactccagtggggaaggcaccaggctcaagaggtggaagaagagacccagactggcaaacgagacatggggttttactggggcttacatacaggggagagagtccgctggtggtgagcgggggcaggagaaaccacaaccggttgcaaaaagcagtttctatagcatttccacttagcaccttcaccctaacaacctccacctggggatcttcattcaacccagacttgggcctggacctttccctttatgtcccgtgttccacagcctggggcctcagatgtttctcatagacaaggaaggactctccaggttggcgactcctggattccctagcttggaagatacattcaggtgtgcctgccacacagggccattctcaggctgtgcttaagttcctgcggtcaggtgcggttaccatacactcctaggtgttcaggccaacagctggtgccccgaaactggtcaggagagaagggacaggccaccgtgaggctgtttgttgtggcggccgctctagaactagtggatccgtccctcactggagctacagacaagaaggtaaaaaacggctgacaaaagaagtcctggtatcctctatgatgggagaaggaaactagctaaagggaagaataaattagagaaaaactggaatgactgaatcggaacaaggcaaaggctataaaaaaaattaagcagcagtatcctcttgggggccccttccccacactatctcaatgcaaatatctgtctgaaacggtccctggctaaactccacccatgggttggccagccttgccttgaccaatagccttgacaaggcaaacttgaccaatagtcttagagtatccagtgaggccaggggccggcggctggctagggatgaagaataaaaggaagcacccttcagcagttccacacactcgcttctggaacgtctgagattatcaataaggacctgcagccaagcttcacgctgccgcaagcactcagggcgcaagggctgctaaaggaagcggaacacgtagaaagccagtccgcagaaacggtgctgaccccggatgaatgtcagctactgggctatctggacaagggaaaacgcaagcgcaaagagaaagcaggtagcttgcagtgggcttacatggcgatagctagactgggcggttttatggacagcaagcgaaccggaattgccagctggggcgccctctggtaaggttgggaagccctgcaaagtaaactggatggctttcttgccgccaaggatctgatggcgcaggggatcaagatctgatcaagagacaggatgaggatcgtttcgcatgattgaacaagatggattgcacgcaggttctccggccgcttgggtggagaggctattcggctatgactgggcacaacagacaatcggctgctctgatgccgccgtgttccggctgtcagcgcaggggcgcccggttctttttgtcaagaccgacctgtccggtgccctgaatgaactgcaggacgaggcagcgcggctatcgtggctggccacgacgggcgttccttgcgcagctgtgctcgacgttgtcactgaagcgggaagggactggctgctattgggcgaagtgccggggcaggatctcctgtcatctcaccttgctcctgccgagaaagtatccatcatggctgatgcaatgcggcggctgcatacgcttgatccggctacctgcccattcgaccaccaagcgaaacatcgcatcgagcgagcacgtactcggatggaagccggtcttgtcgatcaggatgatctggacgaagagcatcaggggctcgcgccagccgaactgttcgccaggctcaaggcgcgcatgcccgacggcgaggatctcgtcgtgacccatggcgatgcctgcttgccgaatatcatggtggaaaatggccgcttttctggattcatcgactgtggccggctgggtgtggcggaccgctatcaggacatagcgttggctacccgtgatattgctgaagagcttggcggcgaatgggctgaccgcttcctcgtgctttacggtatcgccgctcccgattcgcagcgcatcgccttctatcgccttcttgacgagttcttctgagcgggactctggggttcgaaatgaccgaccaagcgacgcccaacctgccatcacgagatttcgattccaccgccgccttctatgaaaggttgggcttcggaatcgttttccgggacgccggctggatgatcctccagcgcggggatctcatgctggagttcttcgcccaccccgggctcgatcccctcgcgagttggttcagctgctgcctgaggctggacgacctcgcggagttctaccggcagtgcaaatccgtcggcatccaggaaaccagcagcggctatccgcgcatccatgcccccgaactgcaggagtggggaggcacgatggccgctttggtcccggatctttgtgaaggaaccttacttctgtggtgtgacataattggacaaactacctacagagatttaaagctctaaggtaaatataaaatttttaagtgtataatgtgttaaactactgattctaattgtttgtgtattttagattccaacctatggaactgatgaatgggagcagtggtggaatgcctttaatgaggaaaacctgttttgctcagaagaaatgccatctagtgatgatgaggctactgctgactctcaacattctactcctccaaaaaagaagagaaaggtagaagaccccaaggactttccttcagaattgctaagttttttgagtcatgctgtgtttagtaatagaactcttgcttgctttgctatttacaccacaaaggaaaaagctgcactgctatacaagaaaattatggaaaaatattctgtaacctttataagtaggcataacagttataatcataacatactgttttttcttactccacacaggcatagagtgtctgctattaataactatgctcaaaaattgtgtacctttagctttttaatttgtaaaggggttaataaggaatatttgatgtatagtgccttgactagagatcataatcagccataccacatttgtagaggttttacttgctttaaaaaacctcccacacctccccctgaacctgaaacataaaatgaatgcaattgttgttgttaacttgtttattgcagcttataatggttacaaataaagcaatagcatcacaaatttcacaaataaagcatttttttcactgcattctagttgtggtttgtccaaactcatcaatgtatcttatcatgtctggatcccccatcaagcttatcgatacccctagagggacagcccccccccaaagcccccagggatgtaattacgtccctcccccgctagggggcagcagcgagccgcccggggctccgctccggtccggcgctccccccgcatccccgagccggcagcgtgcggggacagcccgggcacggggaaggtggcacgggatcgctttcctctgaacgcttctcgctgctctttgagcctgcagacacctggggggatacggggaaaaagctttaggctgaaagagagatttagaatgacagaatcatagaacggcctgggttgcaaaggagcacagtgctcatccagatccaaccccctgctatgtgcagggtcatcaaccagcagcccaggctgcccagagccacatccagcctggccttgaatgcctgcagggatggggcatccacagcctccttgggcaacctgttcagtgcgtcaccaccctctgggggaaaaactgcctcctcatatccaacccaaacctcccctgtctcagtgtaaagccattcccccttgtcctatcaagggggagtttgctgtgacattgttggtctggggtgacacatgtttgccaattcagtgcatcacggagaggcagatcttggggataaggaagtgcaggacagcatggacgtgggacatgcaggtgttgagggctctgggacactctccaagtcacagcgttcagaacagccttaaggataagaagataggatagaaggacaaagagcaagttaaaacccagcatggagaggagcacaaaaaggccacagacactgctggtccctgtgtctgagcctgcatgtttgatggtgtctggatgcaagcagaaggggtggaagagcttgcctggagagatacagctgggtcagtaggactgggacaggcagctggagaattgccatgtagatgttcatacaatcgtcaaatcatgaaggctggaaaagccctccaagatccccaagaccaaccccaacccacccaccgtgcccactggccatgtccctcagtgccacatccccacagttcttcatcacctccagggacggtgacccccccacctccgtgggcagctgtgccactgcagcaccgctctttggagaaggtaaatcttgctaaatccagcccgaccctcccctggcacaacgtaaggccattatctctcatccaactccaggacggagtcagtgagaatggggctctggctcgaggtcgacggtatcgataagcttgatatcgaattcctgcagcccgggggatccactagttctagagcggccgccaccgcggtggagctccaattcgccctatagtgagtcgtattacaattcactggccgtcgttttacaacgtcgtgactgggaaaaccctggcgttacccaacttaatcgccttgcagcacatccccctttcgccagctggcgtaatagcgaagaggcccgcaccgatcgcccttcccaacagttgcgcagcctgaatggcgaatgggacgcgccctgtagcggcgcattaagcgcggcgggtgtggtggttacgcgcagcgtgaccgctacacttgccagcgccctagcgcccgctcctttcgctttcttcccttcctttctcgccacgttcgccggctttccccgtcaagctctaaatcgggggctccctttagggttccgatttagtgctttacggcacctcgaccccaaaaaacttgattagggtgatggttcacgtagtgggccatcgccctgatagacggtttttcgccctttgacgttggagtccacgttctttaatagtggactcttgttccaaactggaacaacactcaaccctatctcggtctattcttttgatttataagggattttgccgatttcggcctattggttaaaaaatgagctgatttaacaaaaatttaacgcgaattttaacaaaatattaacgcttacaatttaggtggcacttttcggggaaatgtgcgcggaacccctatttgtttatttttctaaatacattcaaatatgtatccgctcatgagacaataaccctgataaatgcttcaataatattgaaaaaggaagagtatgagtattcaacatttccgtgtcgcccttattcccttttttgcggcattttgccttcctgtttttgctcacccagaaacgctggtgaaagtaaaagatgctgaagatcagttgggtgcacgagtgggttacatcgaactggatctcaacagcggtaagatccttgagagttttcgccccgaagaacgttttccaatgatgagcacttttaaagttctgctatgtggcgcggtattatcccgtattgacgccgggcaagagcaactcggtcgccgcatacactattctcagaatgacttggttgagtactcaccagtcacagaaaagcatcttacggatggcatgacagtaagagaattatgcagtgctgccataaccatgagtgataacactgcggccaacttacttctgacaacgatcggaggaccgaaggagctaaccgcttttttgcacaacatgggggatcatgtaactcgccttgatcgttgggaaccggagctgaatgaagccataccaaacgacgagcgtgacaccacgatgcctgtagcaatggcaacaacgttgcgcaaactattaactggcgaactacttactctagcttcccggcaacaattaatagactggatggaggcggataaagttgcaggaccacttctgcgctcggcccttccggctggctggtttattgctgataaatctggagccggtgagcgtgggtctcgcggtatcattgcagcactggggccagatggtaagccctcccgtatcgtagttatctacacgacggggagtcaggcaactatggatgaacgaaatagacagatcgctgagataggtgcctcactgattaagcattggtaactgtcagaccaagtttactcatatatactttagattgatttaaaacttcatttttaatttaaaaggatctaggtgaagatcctttttgataatctcatgaccaaaatcccttaacgtgagttttcgttccactgagcgtcagaccccgtagaaaagatcaaaggatcttcttgagatcctttttttctgcgcgtaatctgctgcttgcaaacaaaaaaaccaccgctaccagcggtggtttgtttgccggatcaagagctaccaactctttttccgaaggtaactggcttcagcagagcgcagataccaaatactgtccttctagtgtagccgtagttaggccaccacttcaagaactctgtagcaccgcctacatacctcgctctgctaatcctgttaccagtggctgctgccagtggcgataagtcgtgtcttaccgggttggactcaagacgatagttaccggataaggcgcagcggtcgggctgaacggggggttcgtgcacacagcccagcttggagcgaacgacctacaccgaactgagatacctacagcgtgagctatgagaaagcgccacgcttcccgaagggagaaaggcggacaggtatccggtaagcggcagggtcggaacaggagagcgcacgagggagcttccagggggaaacgcctggtatctttatagtcctgtcgggtttcgccacctctgacttgagcgtcgatttttgtgatgctcgtcaggggggcggagcctatggaaaaacgccagcaacgcggcctttttacggttcctggccttttgctggccttttgctcacatgttctttcctgcgttatcccctgattctgtggataaccgtattaccgcctttgagtgagctgataccgctcgccgcagccgaacgaccgagcgcagcgagtcagtgagcgaggaagcggaagagcgcccaatacgcaaaccgcctctccccgcgcgttggccgattcattaatgcagctggcacgacaggtttcccgactggaaagcgggcagtgagcgcaacgcaattaatgtgagttagctcactcattaggcaccccaggctttacactttatgcttccggctcgtatgttgtgtggaattgtgagcggataacaatttcacacaggaaacagctatgaccatgattacgccaagctcgaaattaaccctcactaaagggaacaaaagctg

**Insulator 44 sequence (CTCF sites are highlighted in yellow):**

ggtaccctgtttccttatctgacctgctttaactgggtaagcttatgaaaagtcttgtgtagaagagaaagggataacagcctgtgctaaatgaggaagctgccttgcctgttcctgctcagtggggtttctggctatactacatcaactgagtcagtgcatcttgactagttcccacacctttctctgaacagagagagtaaagggctcaataagaaaaatacagtttatggtctgtacgttgtattacacgtcatcccttactttctaaaaggcatcttcactgagaaagacatggatttctaaccttacaatctctacaaagttcagaatacataataatcttgaattatgattaactgttagttttgaccaggtcttctggcagacaggtcacatgtgttagtatcacttattcctcaagtgttgatgttagtgtcagcatattaccgatgttccacaaacatttcctgaatgactgttaaacttcctacacattaacgagcctctgcatttttttccagcttccatctatgatttaagtaaactctagttttccacttcttcatattctctctctagatctcaattattgcagtaccactgtccaagggcagaggaggttagctgggcccaggcggagtcaattctctactccccaccctgtgggtgtgttcagccttgtgagccagcatcaggcttgagcacagcagtgctgagtcatgctgagtcatgctgaggcttagggtgtgtggccagatgttttcagctgtgagtgatcagtgctatctgggtctctaggaggaagtccacagggaaggtgaaaagaaaataagtttgctccctgaagaaaacattacttacaccagcattacaatgaaaaggggaccctgccttgctgtgtgacataacctagaatattttattttctagttaaaaattacttctcatgatctttattagaagttataacaaatcattttttagaaatgtaaagtttatttagactcatacctagaagcaacagaatcacacattttagtgtacacacacacacacacacgcacacacacacacatgacttttgttcataaactatgcaattgttaatgaaatgctatttggaatgggcttgaattgcacgggccccccctcgaggtcgacggtatcgatgtactcaccctggcctgtctgacatcggcggccactctcaaaggagaagaggttggagtcgtagccatagcgccgcaagcagagataaggccagcggacggcctcacgccgatgcaggtgcagcaccagctcactctgcgtcagctccatcaccccagagcccagctccaccccctcatcatccacatttgtcacctggaggggagaagacagaagggtcaatgagtgagtccaacaagtgtttgcatgggtccctgagccactgggagacaatctgatgcaggacaagaggtggggaaccaaaggtcacatccgactatcacagactctactgaatgccacataactacatgaagcagtgccatggacccacggcagttctctgggcaaatcctacttctctagtgctgagttttgtcatatggacagtgagggaagaggtaggtagctgacttccaaggtcggtttgttagataacaaatgttagacgaccctagacagcccatgccttagtatccaagggccagatgttaatggggaaggggttcaattgtaaattcaaaatcttgagactgggacaccctgacctaggaaagtcaagcccaaggcagaaccaatgcacatctcctctaccacctccccaactgtgtgcccttactgagggctccctcttctggtgggcaggagaaacatgtgcttcaacatgccccagaatgaggaaggaggaggctccctctacagagaggtgggcaggggagagcacaggtcgacttgggtctgccctcagcttcccacagcaaacagcaaacactgatgttgtgcttactgtgtgctaagcactatttaatttatttatttatttattttttgagatggcatctcgctctgtcacccaggctgaagtgcagcggtgcaatctcagcccactgcaacctccacctcccgggttcaagtgattctcctgcctcagcctcccaagtagctgggattacaggcgtgtgccaccatacctggctaatttttgtatttttagtagagacggggtttcgccatgttggccaggctggtctcgagctcctgacctcaggtgatccgcctgccttggcctccaaaagtgctggtattacaggtgtgagccaccgtgcctggccactaagcactattttaaatactttacgtatattaactcacaaatacagaattttgttttctctaaaaaaaaaagggggggggatatataaagttataaaaatacactgacaacagttccaaaagcaccagcggccgctctagaactagtggatccgtcctaaagggaagaataaattagagaaaaactggaatgactgaatcggaacaaggcaaaggctataaaaaaaattaagcagcagtatcctcttgggggccccttccccacactatctcaatgcaaatatctgtctgaaacggtccctggctaaactccacccatgggttggccagccttgccttgaccaatagccttgacaaggcaaacttgaccaatagtcttagagtatccagtgaggccaggggccggcggctggctagggatgaagaataaaaggaagcacccttcagcagttccacacactcgcttctggaacgtctgagattatcaataaggacctgcagccaagcttcacgctgccgcaagcactcagggcgcaagggctgctaaaggaagcggaacacgtagaaagccagtccgcagaaacggtgctgaccccggatgaatgtcagctactgggctatctggacaagggaaaacgcaagcgcaaagagaaagcaggtagcttgcagtgggcttacatggcgatagctagactgggcggttttatggacagcaagcgaaccggaattgccagctggggcgccctctggtaaggttgggaagccctgcaaagtaaactggatggctttcttgccgccaaggatctgatggcgcaggggatcaagatctgatcaagagacaggatgaggatcgtttcgcatgattgaacaagatggattgcacgcaggttctccggccgcttgggtggagaggctattcggctatgactgggcacaacagacaatcggctgctctgatgccgccgtgttccggctgtcagcgcaggggcgcccggttctttttgtcaagaccgacctgtccggtgccctgaatgaactgcaggacgaggcagcgcggctatcgtggctggccacgacgggcgttccttgcgcagctgtgctcgacgttgtcactgaagcgggaagggactggctgctattgggcgaagtgccggggcaggatctcctgtcatctcaccttgctcctgccgagaaagtatccatcatggctgatgcaatgcggcggctgcatacgcttgatccggctacctgcccattcgaccaccaagcgaaacatcgcatcgagcgagcacgtactcggatggaagccggtcttgtcgatcaggatgatctggacgaagagcatcaggggctcgcgccagccgaactgttcgccaggctcaaggcgcgcatgcccgacggcgaggatctcgtcgtgacccatggcgatgcctgcttgccgaatatcatggtggaaaatggccgcttttctggattcatcgactgtggccggctgggtgtggcggaccgctatcaggacatagcgttggctacccgtgatattgctgaagagcttggcggcgaatgggctgaccgcttcctcgtgctttacggtatcgccgctcccgattcgcagcgcatcgccttctatcgccttcttgacgagttcttctgagcgggactctggggttcgaaatgaccgaccaagcgacgcccaacctgccatcacgagatttcgattccaccgccgccttctatgaaaggttgggcttcggaatcgttttccgggacgccggctggatgatcctccagcgcggggatctcatgctggagttcttcgcccaccccgggctcgatcccctcgcgagttggttcagctgctgcctgaggctggacgacctcgcggagttctaccggcagtgcaaatccgtcggcatccaggaaaccagcagcggctatccgcgcatccatgcccccgaactgcaggagtggggaggcacgatggccgctttggtcccggatctttgtgaaggaaccttacttctgtggtgtgacataattggacaaactacctacagagatttaaagctctaaggtaaatataaaatttttaagtgtataatgtgttaaactactgattctaattgtttgtgtattttagattccaacctatggaactgatgaatgggagcagtggtggaatgcctttaatgaggaaaacctgttttgctcagaagaaatgccatctagtgatgatgaggctactgctgactctcaacattctactcctccaaaaaagaagagaaaggtagaagaccccaaggactttccttcagaattgctaagttttttgagtcatgctgtgtttagtaatagaactcttgcttgctttgctatttacaccacaaaggaaaaagctgcactgctatacaagaaaattatggaaaaatattctgtaacctttataagtaggcataacagttataatcataacatactgttttttcttactccacacaggcatagagtgtctgctattaataactatgctcaaaaattgtgtacctttagctttttaatttgtaaaggggttaataaggaatatttgatgtatagtgccttgactagagatcataatcagccataccacatttgtagaggttttacttgctttaaaaaacctcccacacctccccctgaacctgaaacataaaatgaatgcaattgttgttgttaacttgtttattgcagcttataatggttacaaataaagcaatagcatcacaaatttcacaaataaagcatttttttcactgcattctagttgtggtttgtccaaactcatcaatgtatcttatcatgtctggatcccccatcaagcttatcgatacccctagagggacagcccccccccaaagcccccagggatgtaattacgtccctcccccgctagggggcagcagcgagccgcccggggctccgctccggtccggcgctccccccgcatccccgagccggcagcgtgcggggacagcccgggcacggggaaggtggcacgggatcgctttcctctgaacgcttctcgctgctctttgagcctgcagacacctggggggatacggggaaaaagctttaggctgaaagagagatttagaatgacagaatcatagaacggcctgggttgcaaaggagcacagtgctcatccagatccaaccccctgctatgtgcagggtcatcaaccagcagcccaggctgcccagagccacatccagcctggccttgaatgcctgcagggatggggcatccacagcctccttgggcaacctgttcagtgcgtcaccaccctctgggggaaaaactgcctcctcatatccaacccaaacctcccctgtctcagtgtaaagccattcccccttgtcctatcaagggggagtttgctgtgacattgttggtctggggtgacacatgtttgccaattcagtgcatcacggagaggcagatcttggggataaggaagtgcaggacagcatggacgtgggacatgcaggtgttgagggctctgggacactctccaagtcacagcgttcagaacagccttaaggataagaagataggatagaaggacaaagagcaagttaaaacccagcatggagaggagcacaaaaaggccacagacactgctggtccctgtgtctgagcctgcatgtttgatggtgtctggatgcaagcagaaggggtggaagagcttgcctggagagatacagctgggtcagtaggactgggacaggcagctggagaattgccatgtagatgttcatacaatcgtcaaatcatgaaggctggaaaagccctccaagatccccaagaccaaccccaacccacccaccgtgcccactggccatgtccctcagtgccacatccccacagttcttcatcacctccagggacggtgacccccccacctccgtgggcagctgtgccactgcagcaccgctctttggagaaggtaaatcttgctaaatccagcccgaccctcccctggcacaacgtaaggccattatctctcatccaactccaggacggagtcagtgagaatggggctctggctcgaggtcgacggtatcgataagcttgatatcgaattcctgcagcccgggggatccactagttctagagcggccgccaccgcggtggagctccaattcgccctatagtgagtcgtattacaattcactggccgtcgttttacaacgtcgtgactgggaaaaccctggcgttacccaacttaatcgccttgcagcacatccccctttcgccagctggcgtaatagcgaagaggcccgcaccgatcgcccttcccaacagttgcgcagcctgaatggcgaatgggacgcgccctgtagcggcgcattaagcgcggcgggtgtggtggttacgcgcagcgtgaccgctacacttgccagcgccctagcgcccgctcctttcgctttcttcccttcctttctcgccacgttcgccggctttccccgtcaagctctaaatcgggggctccctttagggttccgatttagtgctttacggcacctcgaccccaaaaaacttgattagggtgatggttcacgtagtgggccatcgccctgatagacggtttttcgccctttgacgttggagtccacgttctttaatagtggactcttgttccaaactggaacaacactcaaccctatctcggtctattcttttgatttataagggattttgccgatttcggcctattggttaaaaaatgagctgatttaacaaaaatttaacgcgaattttaacaaaatattaacgcttacaatttaggtggcacttttcggggaaatgtgcgcggaacccctatttgtttatttttctaaatacattcaaatatgtatccgctcatgagacaataaccctgataaatgcttcaataatattgaaaaaggaagagtatgagtattcaacatttccgtgtcgcccttattcccttttttgcggcattttgccttcctgtttttgctcacccagaaacgctggtgaaagtaaaagatgctgaagatcagttgggtgcacgagtgggttacatcgaactggatctcaacagcggtaagatccttgagagttttcgccccgaagaacgttttccaatgatgagcacttttaaagttctgctatgtggcgcggtattatcccgtattgacgccgggcaagagcaactcggtcgccgcatacactattctcagaatgacttggttgagtactcaccagtcacagaaaagcatcttacggatggcatgacagtaagagaattatgcagtgctgccataaccatgagtgataacactgcggccaacttacttctgacaacgatcggaggaccgaaggagctaaccgcttttttgcacaacatgggggatcatgtaactcgccttgatcgttgggaaccggagctgaatgaagccataccaaacgacgagcgtgacaccacgatgcctgtagcaatggcaacaacgttgcgcaaactattaactggcgaactacttactctagcttcccggcaacaattaatagactggatggaggcggataaagttgcaggaccacttctgcgctcggcccttccggctggctggtttattgctgataaatctggagccggtgagcgtgggtctcgcggtatcattgcagcactggggccagatggtaagccctcccgtatcgtagttatctacacgacggggagtcaggcaactatggatgaacgaaatagacagatcgctgagataggtgcctcactgattaagcattggtaactgtcagaccaagtttactcatatatactttagattgatttaaaacttcatttttaatttaaaaggatctaggtgaagatcctttttgataatctcatgaccaaaatcccttaacgtgagttttcgttccactgagcgtcagaccccgtagaaaagatcaaaggatcttcttgagatcctttttttctgcgcgtaatctgctgcttgcaaacaaaaaaaccaccgctaccagcggtggtttgtttgccggatcaagagctaccaactctttttccgaaggtaactggcttcagcagagcgcagataccaaatactgtccttctagtgtagccgtagttaggccaccacttcaagaactctgtagcaccgcctacatacctcgctctgctaatcctgttaccagtggctgctgccagtggcgataagtcgtgtcttaccgggttggactcaagacgatagttaccggataaggcgcagcggtcgggctgaacggggggttcgtgcacacagcccagcttggagcgaacgacctacaccgaactgagatacctacagcgtgagctatgagaaagcgccacgcttcccgaagggagaaaggcggacaggtatccggtaagcggcagggtcggaacaggagagcgcacgagggagcttccagggggaaacgcctggtatctttatagtcctgtcgggtttcgccacctctgacttgagcgtcgatttttgtgatgctcgtcaggggggcggagcctatggaaaaacgccagcaacgcggcctttttacggttcctggccttttgctggccttttgctcacatgttctttcctgcgttatcccctgattctgtggataaccgtattaccgcctttgagtgagctgataccgctcgccgcagccgaacgaccgagcgcagcgagtcagtgagcgaggaagcggaagagcgcccaatacgcaaaccgcctctccccgcgcgttggccgattcattaatgcagctggcacgacaggtttcccgactggaaagcgggcagtgagcgcaacgcaattaatgtgagttagctcactcattaggcaccccaggctttacactttatgcttccggctcgtatgttgtgtggaattgtgagcggataacaatttcacacaggaaacagctatgaccatgattacgccaagctcgaaattaaccctcactaaagggaacaaaagctg
